# Baselining physiological parameters in three muscles across three equine breeds. What can we learn from the horse?

**DOI:** 10.3389/fphys.2024.1291151

**Published:** 2024-02-07

**Authors:** Carmen Vidal Moreno de Vega, Constance de Meeûs d’Argenteuil, Berit Boshuizen, Lorie De Mare, Yannick Gansemans, Filip Van Nieuwerburgh, Dieter Deforce, Klara Goethals, Ward De Spiegelaere, Luc Leybaert, Elisabeth-Lidwien J.M.M. Verdegaal, Cathérine Delesalle

**Affiliations:** ^1^ Department of Translational Physiology, Infectiology and Public Health, Research Group of Comparative Physiology, Faculty of Veterinary Medicine, Ghent University, Merelbeke, Belgium; ^2^ Wolvega Equine Hospital, Oldeholtpade, Netherlands; ^3^ Department of Pharmaceutics, Laboratory of Pharmaceutical Biotechnology, Ghent University, Ghent, Belgium; ^4^ Biometrics Research Center, Ghent University, Ghent, Belgium; ^5^ Department of Morphology, Imaging, Orthopedics, Rehabilitation and Nutrition, Faculty of Veterinary Medicine, Ghent University, Merelbeke, Belgium; ^6^ Department of Basic and Applied Medical Sciences, Faculty of Medicine and Health Sciences, Ghent University, Ghent, Belgium; ^7^ Thermoregulation Research Group, School of Animal and Veterinary Sciences, Roseworthy Campus, University of Adelaide, Roseworthy, SA, Australia

**Keywords:** locomotor muscle, posture muscle, Warmblood horse, Friesian horse, Standardbred horse, baseline metabolism, acylcarnitine, muscle fiber features

## Abstract

Mapping-out baseline physiological muscle parameters with their metabolic blueprint across multiple archetype equine breeds, will contribute to better understanding their functionality, even across species.

**Aims:** 1) to map out and compare the baseline fiber type composition, fiber type and mean fiber cross-sectional area (fCSA, mfCSA) and metabolic blueprint of three muscles in 3 different breeds 2) to study possible associations between differences in histomorphological parameters and baseline metabolism.

**Methods:** Muscle biopsies [*m. pectoralis* (PM), *m. vastus lateralis* (VL) and *m. semitendinosus* (ST)] were harvested of 7 untrained Friesians, 12 Standardbred and 4 Warmblood mares. Untargeted metabolomics was performed on the VL and PM of Friesian and Warmblood horses and the VL of Standardbreds using UHPLC/MS/MS and GC/MS. Breed effect on fiber type percentage and fCSA and mfCSA was tested with Kruskal-Wallis. Breeds were compared with Wilcoxon rank-sum test, with Bonferroni correction. Spearman correlation explored the association between the metabolic blueprint and morphometric parameters.

**Results:** The ST was least and the VL most discriminative across breeds. In Standardbreds, a significantly higher proportion of type IIA fibers was represented in PM and VL. Friesians showed a significantly higher representation of type IIX fibers in the PM. No significant differences in fCSA were present across breeds. A significantly larger mfCSA was seen in the VL of Standardbreds. Lipid and nucleotide super pathways were significantly more upregulated in Friesians, with increased activity of short and medium-chain acylcarnitines together with increased abundance of long chain and polyunsaturated fatty acids. Standardbreds showed highly active xenobiotic pathways and high activity of long and very long chain acylcarnitines. Amino acid metabolism was similar across breeds, with branched and aromatic amino acid sub-pathways being highly active in Friesians. Carbohydrate, amino acid and nucleotide super pathways and carnitine metabolism showed higher activity in Warmbloods compared to Standardbreds.

**Conclusion:** Results show important metabolic differences between equine breeds for lipid, amino acid, nucleotide and carbohydrate metabolism and in that order. Mapping the metabolic profile together with morphometric parameters provides trainers, owners and researchers with crucial information to develop future strategies with respect to customized training and dietary regimens to reach full potential in optimal welfare.

## Introduction

Translating the physiological meaning of specific morphophysiological properties such as muscle fiber type composition and mean muscle fiber cross-sectional area of specific muscle groups to their individual metabolic blueprint remains a challenge, although such insights would contribute significantly to a better understanding of the complex adaptations that muscle tissue can show in response to all kinds of stimuli. Within the muscles, there is an enormous diversity of energy cycles that can be either up or downregulated in answer to different types of challenges, either imposed in an acute, *versus* a more long-term fashion. All these adaptations start from a certain baseline, which is often overlooked when extrapolating study results from one species to another or from one breed to another. Some adaptations mainly rely on shifts in the preference and sequence of use of, for example, reserve fuels that are stored within the muscle cell itself, for instance, glycogen, which requires room for storage capacity within the muscle fiber. Other energy cycles furnish themselves mainly via fuels that are being supplied on demand. The latter option obviously requires thorough adaptation of capillarization and reshaping towards a smaller muscle fiber size in order to allow for swift diffusion of candidate fuels (Giacomello et al., 2020; Betz et al., 2021; de Meeûs d’Argenteuil et al., 2021b).

It is well known that the muscular compartment plays a pivotal role in the energy metabolism of animals and humans. It encompasses on average almost 40% of the total body mass in most mammals (Klein, 2020). The muscle compartment is composed out of a wide variety of muscle groups, all of which have their specific main role (for example maintenance of posture *versus* realization of locomotion or a mixture of both) and on top of that, this compartment is highly malleable, continuously adapting itself in answer to growth, activity and dietary habits of its host (Leckey et al., 2018; Moro et al., 2019; Robertson and McClelland, 2019). Several different morphophysiological features, that are specific to the muscular compartment, and a longitudinal follow-up of the evolution of those features can be used to obtain a view on not only basal metabolic activity, but also adaptation of the muscular machinery in response to certain challenges, such as training. Some of these features can be visualized histochemically, such as muscle fiber type composition, the fiber specific and mean cross sectional area (fCSA and mfCSA), and the degree of capillarization and mitochondrial density present within a certain muscle (Moro et al., 2019; de Meeûs d’Argenteuil et al., 2021b; van Boom et al., 2022). Other factors can be monitored biochemically, for instance the activity of certain enzyme systems that catalyze different energy cycles (Warren et al., 2014; Srisawat et al., 2017; Moro et al., 2019). Another approach is the assessment of levels of specific metabolites that mirror activity of different energy cycles, both in the aerobic, or in the anaerobic window. These metabolites can be analyzed in either a targeted way (i.e.: involving only a limited amount of pre-selected metabolites) (Jacob et al., 2018; Cao et al., 2020) as well as in an untargeted way (Klein et al., 2020; Fulghum et al., 2022).

When an individual is subjected to a certain training load, muscle plasticity will occur, leading to shifts in one or more of the above-mentioned parameters (Tyler et al., 1998; Larson et al., 2019; Moro et al., 2019; de Meeûs d’Argenteuil et al., 2021a; de Meeûs d’Argenteuil et al., 2021b). From a comparative viewpoint, it is key to keep in mind that important baseline differences exist between these parameters when comparing animal species and within the same animal species, when comparing breeds (Snow and Guy, 1980; Kohn et al., 2011; Schiaffino and Reggiani, 2011; Rivero and Hill, 2016). Especially, when extrapolating muscular plasticity study results from one species to another, it is important to remember that these differences determine the baseline from which the muscular adaptation begins (Ringmark et al., 2016; Nyerges-Bohák et al., 2021). It is also more than likely that the basic pattern from which the adaptation starts off also has an impact on the morphological metamorphosis that is being realized and the timeline that this adaptation follows. Nevertheless, comparing muscle fiber morphometrics and metabolic output between different breeds can provide valuable insights into the physiological and performance characteristics.

For example, fCSA can serve as an indicator of the main metabolic profile of a certain muscle fiber (Van Wessel et al., 2010; Moro et al., 2019). Several studies have shown that the fCSA is associated with the degree of acute force generation and power output capacity of a muscle and have suggested that a larger fCSA allows for accumulation of more reserve fuel molecules, enabling the muscle fiber to generate explosive bouts of exercise and force (Miller et al., 2015). A smaller fCSA is, in turn, associated with a higher oxidative capacity, since the diffusion distance for oxygen and metabolites is shorter, allowing for execution of low intensity exercise for extended time periods (Van Wessel et al., 2010).

We have previously shown that shifts in metabolic fingerprint and shifts in fCSA and capillarization precede shifts in fiber type composition and mitochondrial density in answer to training, meaning that metabolic fingerprint, muscle fCSA and muscle fiber capillarization evolution need to be viewed as early biomarkers for muscular metabolic adaptation (de Meeûs d’Argenteuil et al., 2021b). We have also shown that, in horses, in answer to aerobic training, the muscular machinery models itself towards an optimal smaller individual muscle fCSA to receive and process fuels that can be swiftly delivered by the circulatory system (de Meeûs d’Argenteuil et al., 2021b). With that respect, gut microbiome-derived metabolites showed important upregulation after training in Standardbred horses. It is more than feasible that the circulatory system may act as a conduit to provide a continuous flow of these gut microbiome-derived metabolites towards the muscle fibers being trained in the aerobic window (Klein et al., 2020; Przewłócka et al., 2020; de Meeûs d’Argenteuil et al., 2021a).

In humans, a wide array of studies has been performed in an attempt to link fiber type composition with genetics, ethnic background and athletic performance capacity in certain sports disciplines (Harpalani, 2017; Ahmad Yusof and Che Muhamed, 2021; Saito et al., 2022). However, due to the genetic diversity within the human population, at this point it is still quite challenging to directly link certain muscular physiological parameters to a specific athletic performance capacity type (Ahmad Yusof and Che Muhamed, 2021; Saito et al., 2022). Researchers also aim to identify a certain baseline muscle fiber type composition pattern and mfCSA as the ultimate guideline to forecast outperformance of novice athletes in certain sports disciplines (Lievens et al., 2020; Lievens et al., 2021b; Lievens et al., 2021a; Lievens et al., 2022; Hall et al., 2021).

In horses, the genetic background is governed by, most often closed, studbooks, which crucially adds to the homogeneity of that study population (Cosgrove et al., 2020; Marín Navas et al., 2021). Because of this, equine pedigrees and origins of familial offspring have a well-defined provenance and homogeneous genetic basis. Each specific horse breed, governed by its specific studbook, is the result of hundreds of years of selection to achieve ultimate performance levels in certain desired areas such as speed, strength, endurance, etc. Keeping this in mind, mapping out multiple baseline muscular physiological parameters in different archetype horse breeds such as endurance *versus* racing can importantly add to the identification of pivotal morphophysiological biomarkers across species. Several equine studies have been performed concerning mapping out breed-specific fiber type composition. Table 1 provides an overview of baseline representation of the different muscle fiber types in different horse breeds, in the *m. gluteus medius* (GM), which is an important locomotor muscle in the hindquarters and the most studied muscle in the equine species.

**TABLE 1:** Overview of baseline muscle fiber type composition in different horse breeds in the *m. gluteus medius*.

Breed	Main feature(s)	% Type I fibers	% Type IIA and IIA/IIX fibers	% Type IIX fibers	References
Quarter Horse 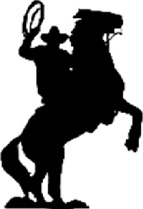	Sprinting capacity	7–8	30–48	45–62	Snow and Guy, 1980; Valberg et al., 2022
Thoroughbred 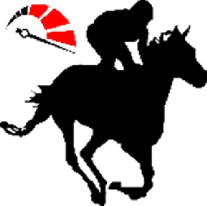	Sprinting capacity	12–29	39–51	32–41	Snow and Guy, 1980; Valberg, 1987; López-Rivero et al., 1989
Standardbred 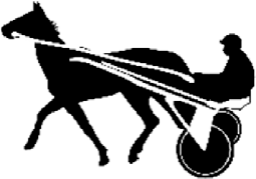	Harness racing	23	47	30	Valberg (1987)
	Endurance capacity				
Arabian 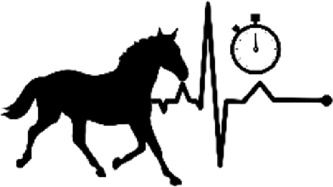	Long distance racing	14–26	42–48	28–38	Snow and Guy, 1980; López-Rivero et al., 1989; Rivero et al., 1995
	Endurance capacity				
Andalusian 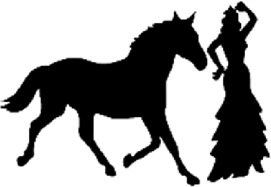	Dressage	26–35	37–41	28–32	López-Rivero et al., 1989, 1991; Rivero et al., 1995
Haflinger		32–40	37–41	26–27	Lindner et al. (2013)
Heavy hunter 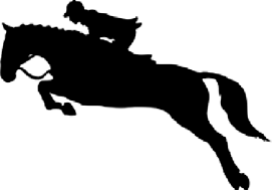	Jumping capacity	31	37	32	Snow and Guy (1980)
Half breed draught horse 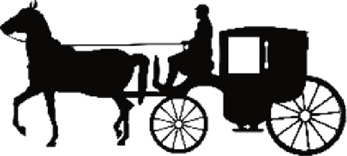	Carriage driving	47	24	29	Islas et al. (1997)
	Power capacity				
Dutch Warmblood	Dressage	26	58	16	van Ginneken et al. (2006)
	Jumping				
	Eventing				
Warmblood (general) 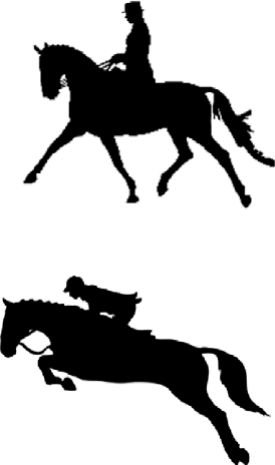	Dressage	31–39	33–37	23–35	Valberg et al. (2022)
	Jumping				
	Eventing				
Belgian draft 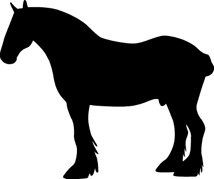	Carriage driving	16	25	60	Firsham et al. (2008)
	Power capacity				
Pony		23	40	37	Snow and Guy (1980)

As mentioned previously, also parameters such as fCSA and mfCSA are of importance for metabolic and performance capacity typology (Dechick et al., 2020; de Meeûs d’Argenteuil et al., 2021b). In horses, breed differences have been reported with that respect. For instance, at baseline, Andalusian horses, which are well known for powerful equestrian activities of rather short duration, such as dressage, show jumping and driving, have a significantly larger fCSA across all muscle fiber types when compared to Dutch Warmblood horses, Arabian and Thoroughbred horses (Rivero and Diz, 1992; Serrano and Rivero, 2000; Rietbroek et al., 2007). Similarly, the Greyhound, which is the canine archetype of acceleration power, has a larger mfCSA when compared to a Yorkshire Terrier (van Boom et al., 2023).

A final important musculo-physiological factor that needs to be determined is the metabolic fingerprint of a certain muscle group. Indeed, the baseline metabolic fingerprint of an animal species or specific breed within that species needs to be viewed as an important performance typology indicator. The patchwork of muscle fiber types harbored within each muscle group determines the general metabolic profile of a muscle group, which can be identified using untargeted metabolomics. By combining this analysis method with more conventional techniques such as muscle fiber typing and CSA determination, and possibly finding associations between all these baseline parameters, it becomes feasible to obtain novel insights into the muscular metabolic circuitry; better understand the pathophysiological course of certain neuro-muscular diseases and develop species and breed specific training and dietary management protocols. Performing such studies can also further furnish knowledge to, for example, optimize the newly available techniques used for the prospective classification of individuals into specific sports categories (Lievens et al., 2020; Lievens et al., 2021b; Lievens et al., 2022).

As a final remark, it is important to keep in mind which muscle groups have been sampled when comparing and extrapolating study results between species and between breeds. Depending on the main physiological role of a certain muscle group, such as locomotion *versus* maintenance of posture, physiological muscular features will differ. Moreover, involving multiple muscle groups covering different biomechanical roles adds to better understanding of complex physiological relations between biomarkers.

To conclude, comparing breed specific representation of muscle fiber morphometrics and metabolic characteristics of muscle tissues can reveal information about the predominant energy systems used by different breeds. Some breeds may rely more on glycolytic pathways, which are efficient for short bursts of energy, while others may have a higher oxidative capacity, suited for sustained, aerobic activities. Knowledge of metabolic profiles can help in tailoring nutrition programs for different breeds. Some breeds may require diets that support high-intensity, short-duration activities, while others might benefit from diets promoting endurance and sustained effort. In summary, comparing muscle fiber morphometrics and metabolic properties between breeds provides a holistic understanding of their physiological characteristics, helping breeders, researchers, and practitioners make informed decisions regarding breeding, training, and management practices for optimal performance and health.

The horse as a research species with its plethora of purely bred breeds, each with their own agility purpose and homogenous offspring, can contribute to better understanding the association between different muscular morphophysiological parameters.

### Aim and purpose

The aims of the current study were to map out and compare the baseline fiber type composition, fiber type and mean fiber cross-sectional area (fCSA and mfCSA respectively) and untargeted metabolic profile in three specific muscles (*m. pectoralis* (PM), *m. vastus lateralis* (VL) and *m. semitendinosus* (ST)), which represent different roles (i.e.: posture vs. locomotion), in three different equine breeds and to explore possible associations between these muscular physiological parameters. To the best of our knowledge, this is the first study to choose such an approach. For this purpose, three importantly differing horse breeds were selected, and three different muscle groups with varying main physiological functions were sampled.

## Materials and methods

**FIGURE 1:**
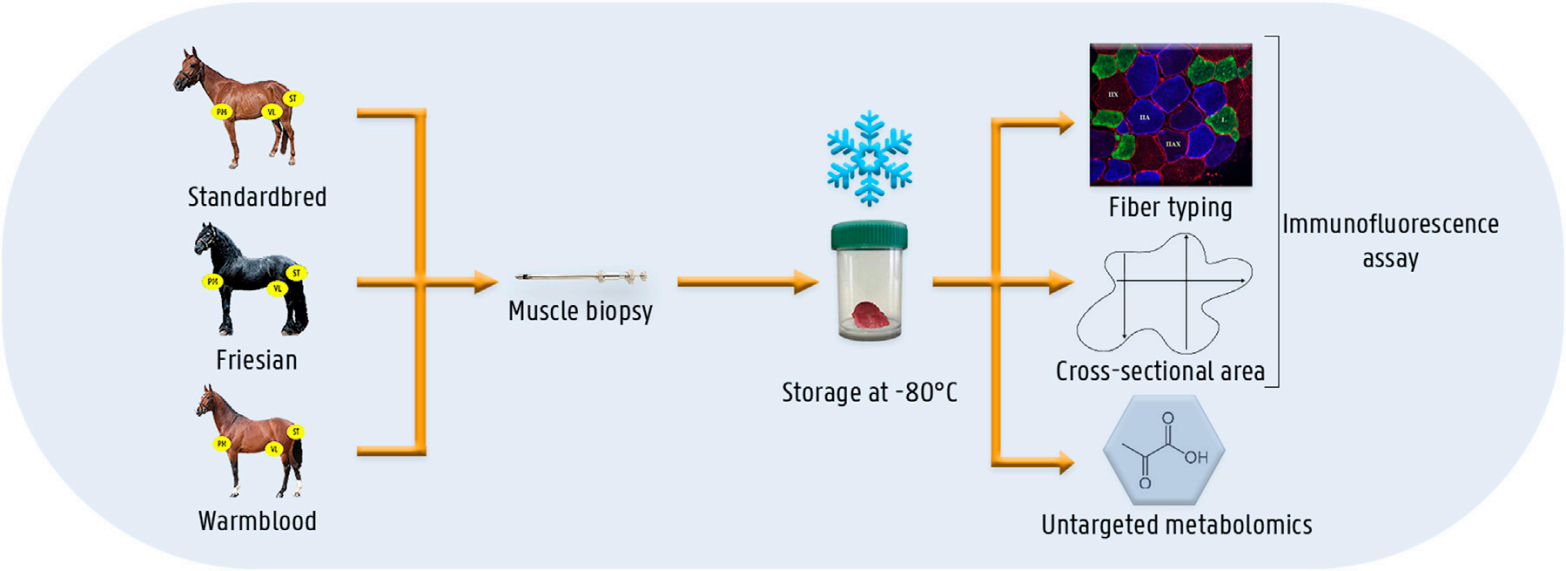
Schematic overview of the materials and methods. Muscle biopsies were taken from three different muscles (i.e.,: *m. pectoralis*, *m. vastus lateralis* and *m. semitendinosus*) of three different horse breed populations (i.e.,: Standardbred, Friesian, Warmblood). Muscle biopsies were instantly snap-frozen until analysis. The different laboratory techniques involved an immunofluorescent assay for fiber type and cross-sectional area determination and untargeted metabolomics.

For this study, a group of healthy untrained Friesian horses (*n* = 7; age range 2.5–3.5 years; 4 ♀ and 3 ♂), Standardbred horses (*n* = 12; age 3–5 years, all ♀) and Warmblood horses (*n* = 4; age 4–5 years, all ♀) was enrolled (Figure 1). Standardbred, Friesian and Warmblood horses were comparable in body condition score (5.5 ± 0.70) and body weight (446.5 ± 30.22 kg). The study was preceded by 2 weeks of acclimation. The horses were not involved in either training or competition during the 4 months prior to this study. Horses were stabled at the same premises for each breed. The same concentrate feed was used throughout the study in all horses. Horses were fed concentrate feed twice a day, at 8 a.m. and 8 p.m. and had *ad libitum* access to roughage. The aforementioned protocol was applied during 4 consecutive weeks, after which muscle biopsies were harvested. Horses were housed in individual boxes with straw bedding. Turn-out was provided on paddocks 2 h a day. Vital signs were recorded twice a day and were within normal limits throughout the entire study: rectal temperature, respiratory rate, heart rate, capillary refill time and color of mucous membranes, appetite and fecal output and consistency. This study was approved by the Animal Ethics Committee of the Ghent University (EC 2016/40) and by the Centrale Commissie Dierproeven, The Hague, Netherlands (AVD262002015144).

### Muscle biopsies

Muscle biopsies were harvested from the PM, VL and the ST as previously described (de Meeûs d’Argenteuil et al., 2021b). Briefly, the horses were sedated with detomidine (10 μg/kg bwt) (Detogesic^®^, Vetcare, Finland) and butorphanol (20 μg/kg bwt) (Butomidor, Richter Pharma AG, Wels, Austria). The area was clipped, shaved and subsequently surgically disinfected. Local anesthetic ointment was applied (Emla^®^ 5%, Astra-Zeneca, Rueil-Malmaison, France). After 10 min, a local anesthetic solution (Lidocaine Hydrochloride^®^, Braun, Germany) was injected subcutaneously and a small stab incision was made with a surgical blade number 11 midway between origin and insertion of each muscle, as previously described (de Meeûs d’Argenteuil et al., 2021b). Subsequently, a 14G Bergström needle was inserted into the muscle, until a depth of 4 cm was reached on each occasion. Samples were taken under suction pressure, to obtain a total of approximately 120 mg of muscle tissue, which was then divided into three portions: one portion was embedded in Tissue-Tek^®^ OCT compound (Sakura Finetek, Torrance, CA) and was immediately snap-frozen in isopentane in liquid nitrogen and stored at −80°C until processed for muscle fiber typing and fiber CSA assessment. The remaining portions were immediately snap-frozen in liquid nitrogen and stored at −80°C until processed for untargeted metabolomics.

### Muscle fiber typing, assessment of fiber CSA and mean fiber CSA

Both the muscle fiber typing and the untargeted metabolomics were performed as previously described (de Meeûs d’Argenteuil et al., 2021a; de Meeûs d’Argenteuil et al., 2021b). Briefly, cryosections of 8 µm were cut from the Tissue-Tek^®^ embedded samples from the PM, VL and ST and were placed on Thermo Scientific^™^ SuperFrost Plus^™^ Adhesion slides. The sections were air-dried and blocked for 120 min in 1% BSA in PBS solution. Thereafter, the slides were incubated overnight with the primary antibodies for the different myosin heavy chain subtypes present in respectively type I, type IIA and type IIX fibers, and the sarcolemma (respectively BA-D5, DSHB, RRID:AB_2235587; SC-71, DSHB, RRID:AB_2147165; 6H1, DSHB, RRID:AB_1157897 and laminin, Thermo Fisher Scientific Cat: PA1-36119, RRID:AB_2133620) (Latham and White, 2017). After rinsing the slides 5 consecutive times during 5 min in PBS, they were incubated with the secondary antibodies for 1 h at room temperature for type I, IIA, IIX and sarcolemma (respectively: Alexa fluor 488 goat anti-mouse IgG2b, Thermo Fisher Scientific Cat: A-21141, RRID:AB_2535778; Alexa fluor 350 goat anti-mouse IgG1, Thermo Fisher Scientific Cat: A21120, RRID:AB_2535763; Alexa fluor 594 goat anti-mouse IgM, Thermo Fisher Scientific Cat: A-21044, RRID:AB_2535713; Alexa fluor 568 goat anti-rabbit IgG, Thermo Fisher Scientific Cat: A-11011, RRID:AB_143157). Fluorescent mounting medium (Dako, Agilent, S3023) was then applied to the slides. The sections were visualized with a Zeiss Palm Micro Beam fluorescence microscope and pictures were taken with the Zen Blue Pro1 Software (Zeiss). The fibers were classified as either type I (green), type IIA (blue), type IIX (red) or as hybrid type when staining of more than one myosin heavy chain type was visualized. The hybrid type IIA/IIX (IIAX) was included in the analysis, but not the type I/IIA since it was only sporadically identified (<1%).

The mean CSA of each fiber type, will be annotated as fCSA, as well as the overall mean CSA across all muscle fiber types, called henceforth mfCSA, were determined by means of an automated software analysis program (Image Pro v10 analyzer software, Media Cybernetics Inc., Rockville, United States), which had previously been validated for that purpose (de Meeûs d’Argenteuil et al., 2021a; de Meeûs d’Argenteuil et al., 2021b).

### Execution of untargeted metabolomics

Untargeted metabolomics analysis was performed on the VL and PM samples of Friesian and Warmblood horses and the VL of Standardbred horses as previously described (de Meeûs d’Argenteuil et al., 2021b; Metabolon Inc., Durham, NC). Briefly, Ultra High Performance Liquid chromatography/Mass Spectrometry/Mass Spectrometry (UHPLC/MS/MS) and Gas chromatography/Mass Spectrometry (GC/MS) were performed (Evans et al., 2009; Dehaven et al., 2010). Two columns, C18 and a hydrophilic interaction liquid chromatography (HILIC) column, were used. The extracts were divided into five fractions: two for analysis by two separate reverse phase (RP)/UPLC-MS/MS methods with positive ion mode electrospray ionization (ESI), one for analysis by RP/UPLC-MS/MS with negative ion mode ESI, one for analysis by Hydrophilic Interaction Ultra Performance Liquid Chromatography/Mass Spectrometry/Mass Spectrometry (HILIC/UPLC-MS/MS) with negative ion mode ESI and one sample was reserved as a backup. All methods utilized a Waters ACQUITY ultra-performance liquid chromatography (UPLC) and a Thermo Scientific Q Exactive high resolution/accurate mass spectrometer interfaced with a heated electrospray ionization (HESI-II) source and Orbitrap mass analyzer operated at 35,000 mass resolution.

### Statistical analysis

Statistical analysis for fiber type composition, fCSA and mfCSA was performed in R (R version 3.6, Core Team, 2019). A Kruskal-Wallis test at a significance level of 0.05 was used to assess the overall effect of breed on percentage and both fCSA and mfCSA. When the Kruskal-Wallis test was significant, the breeds were compared two-by-two, for a total of three comparisons, for each muscle parameter using a Wilcoxon rank-sum test at a significance level of 0.017 to account for multiple comparisons, using Bonferroni to correct for multiple comparisons.

For the metabolomics analysis, data were analysed using R (version 3.6). To investigate the differential presence of metabolites based on the concentration found across the three breeds in the VL and between Warmblood and Friesian horses in the PM, a Kruskal-Wallis test was used for group comparisons and a Wilcoxon rank-sum test for pairwise comparisons, for a total of three comparisons. Significance was set at *p* < 0.05. For the fold difference (FD), the mean concentration of each metabolite for each breed was divided by the mean concentration of the same metabolite in another breed. The differences between breeds in baseline metabolism are primarily discussed throughout the manuscript based on the following super pathways and on occasion focusing on their specific metabolites: lipids, carbohydrates, energy, amino acids, nucleotides and xenobiotics. A Spearman correlation was performed in order to explore the association between each of the metabolites found in each breed and the three muscular physiological parameters: fiber type composition, fCSA and mfCSA. The false discovery rate was addressed with the Benjamini-Hochberg procedure.

## Results

### Breed differences in muscle fiber type composition in the PM, VL and ST muscle

**FIGURE 2:**
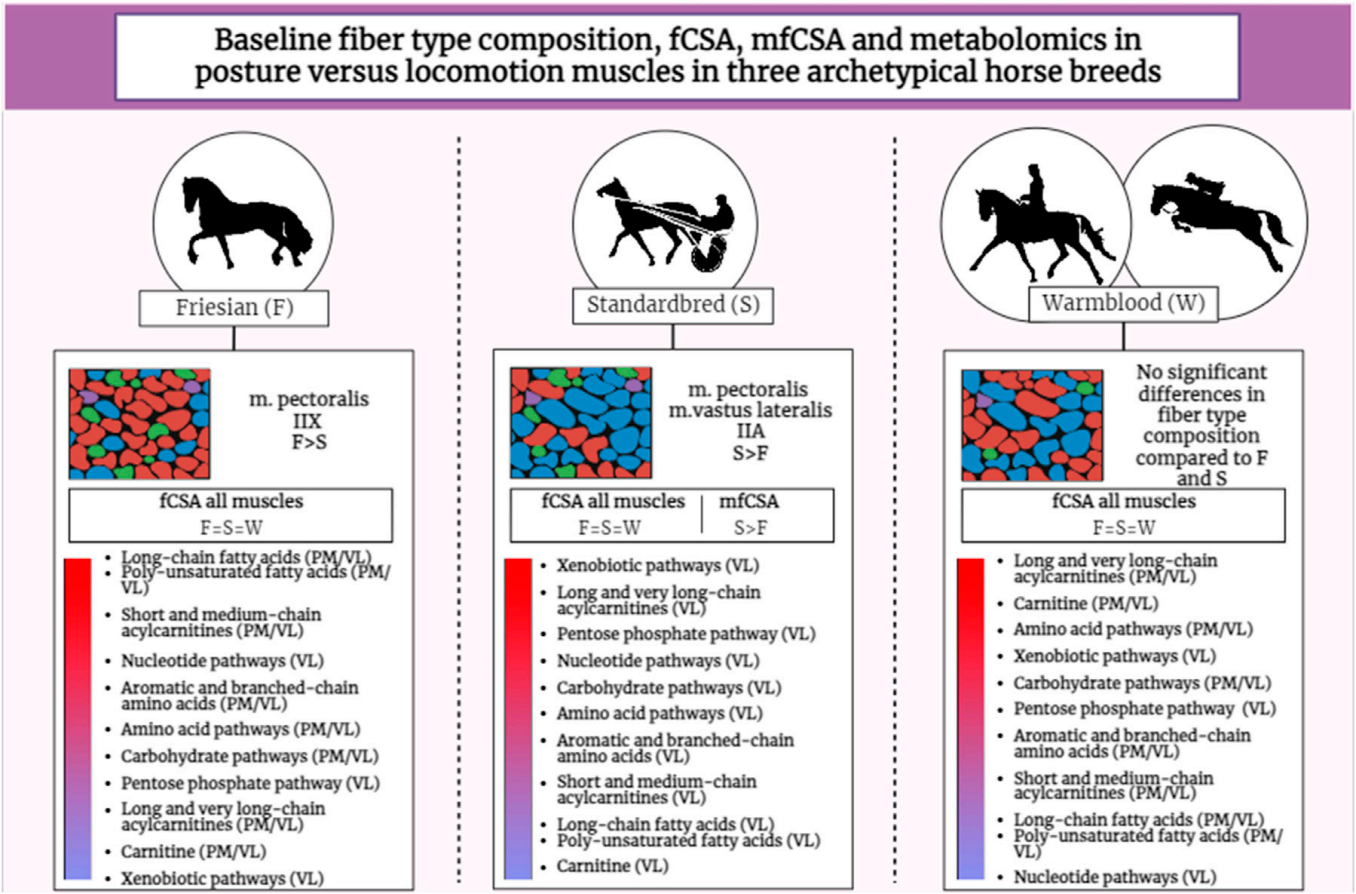
Graphical abstract illustrating the results of the study across the three studied horse breeds. The gradient bar represents the abundance of metabolites per energy cycle (based on heatmap results) from high (red) to low (blue) across breeds. Friesian (F); Standardbred (S); Warmblood (W); *m. pectoralis* (PM); *m. vastus lateralis* (VL); Fiber cross-sectional area (fCSA).

**FIGURE 3:**
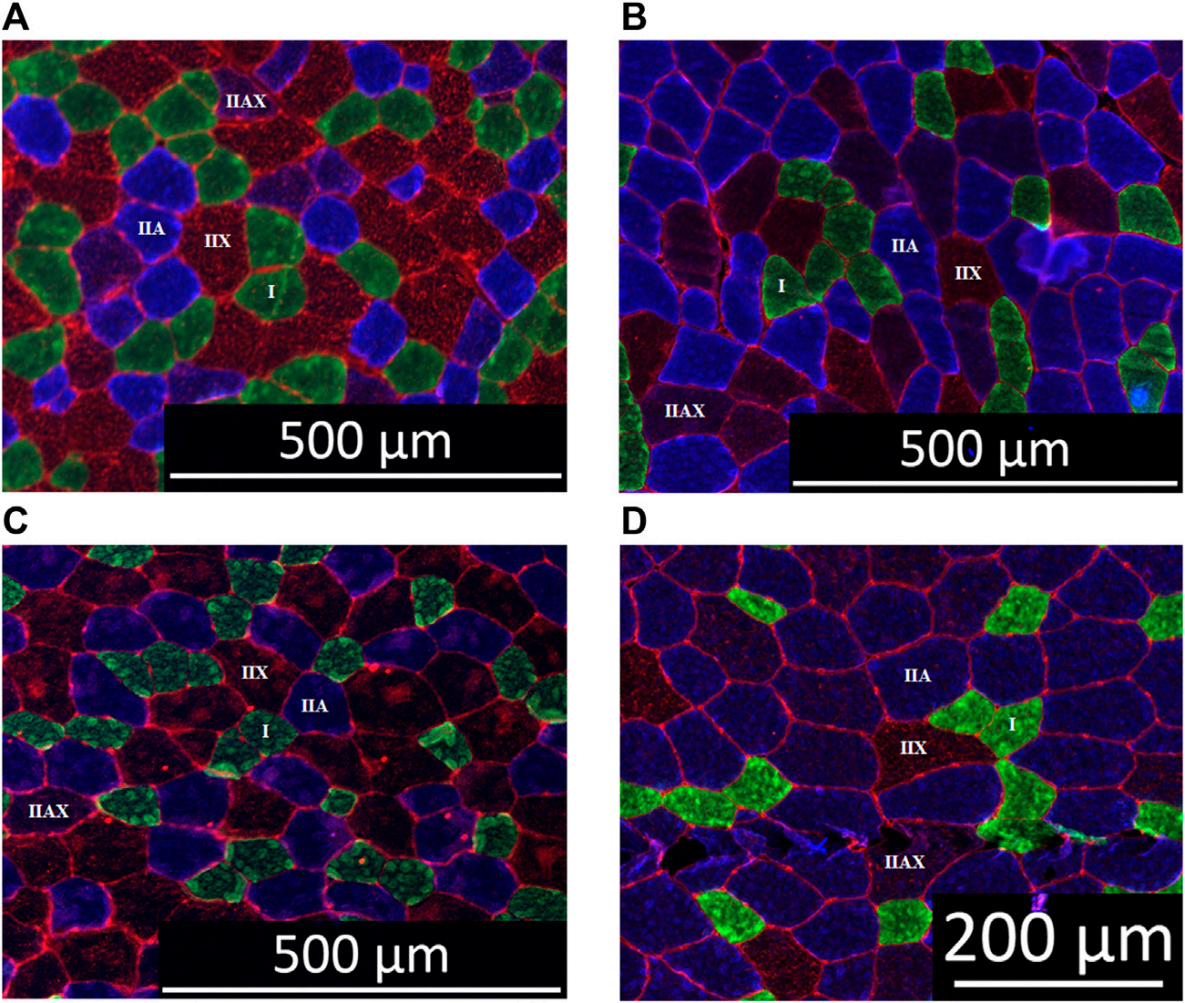
Immunohistochemical staining of equine muscle: type I (green), type IIA (blue), type IIX (red) and type IIAX (blue/red) fibers. Panel **(A)** Friesian horse, m. pectoralis, scale bar 500 µm. Panel **(B)** Standardbred horse, m. pectoralis, scale bar 200 µm. Panel **(C)** Friesian horse, *m. vastus lateralis*, scale bar 500 µm. Panel **(D)** Standardbred horse, *m. vastus lateralis*, scale bar 500 µm.

Significant differences (Table 2) were seen in the fiber type composition between breeds and between muscle groups, with the exception of the ST, which showed a similar fiber type composition across all studied breeds and thus was the least discriminative muscle between breeds; as opposed to the PM (Figures 2A, B) and the VL muscle (Figures 2C, D). Overall, representation of type I fibers was alike for all studied muscles and across studied breeds. The most pronounced differences in muscle fiber type representation were seen for type IIA and IIX fibers. The baseline fiber type composition profile for the three muscle groups in each horse breed is represented in Figures 3 4. A summary of all the results in this study can be found in Figure 5.

**TABLE 2:** Median percentage and ranges of type I, IIA, IIAX and IIX fibers for Friesian, Standardbred and Warmblood horses in the *m. pectoralis* (PM), *m. vastus lateralis* (VL) and *m. semitendinosus* (ST). Significant findings are highlighted in red.

		PM			VL			ST		
		Friesian	Standardbred	Warmblood	Friesian	Standardbred	Warmblood	Friesian	Standardbred	Warmblood
Percentage of fiber types (%)	Type I	25.62 (19.56–32.56)	24.68 (21.09–37.5)	26.07 (25.97–26.18)	25.17 (21.35–41.18)	20.39 (3.54–30.16)	25.67 (18–33.33)	13.17 (7.46–31.21)	13.59 (0.13–30.43)	12.47 (6.25–18.69)
	Type IIA	38.37 (20–44.02)	52.82 (39.34–68.79)^*^	29.16 (21.72–36.53)	35.29 (30.62–45.76)	54.87 (42.36–66.27)^*^	55.38 (48.68–62.09)	32.63 (29.48–52.77)	46.73 (24.75–71.13)	36.64 (32.94–40.34)
	Type IIAX	9.59 (4.01–11.92)	6.94 (1.4–12.47)	11 (10.92–11.09)	7.81 (2.98–13.45)	8.41 (2.08–14.44)	11 (10.92–11.09)	9.5 (3.05–12.05)	5.4 (0.84–18.66)	10.6 (8.61–12.5)
	Type IIX	27.78 (19.78–37.59)^**^	16.63 (2.13–28.81)	33.8 (26.37–41.23)	29.83 (10.1–39.95)	17.5 (4.73–33.19)	12 (9.48–14.47)	39.74 (19.15–52.55)	34.52 (21.24–62.37)	40.34 (39.76–40.91)

^*^Significantly higher value compared to Friesian horses; ^**^Significantly higher value compared to Standardbred horses.

**FIGURE 4:**
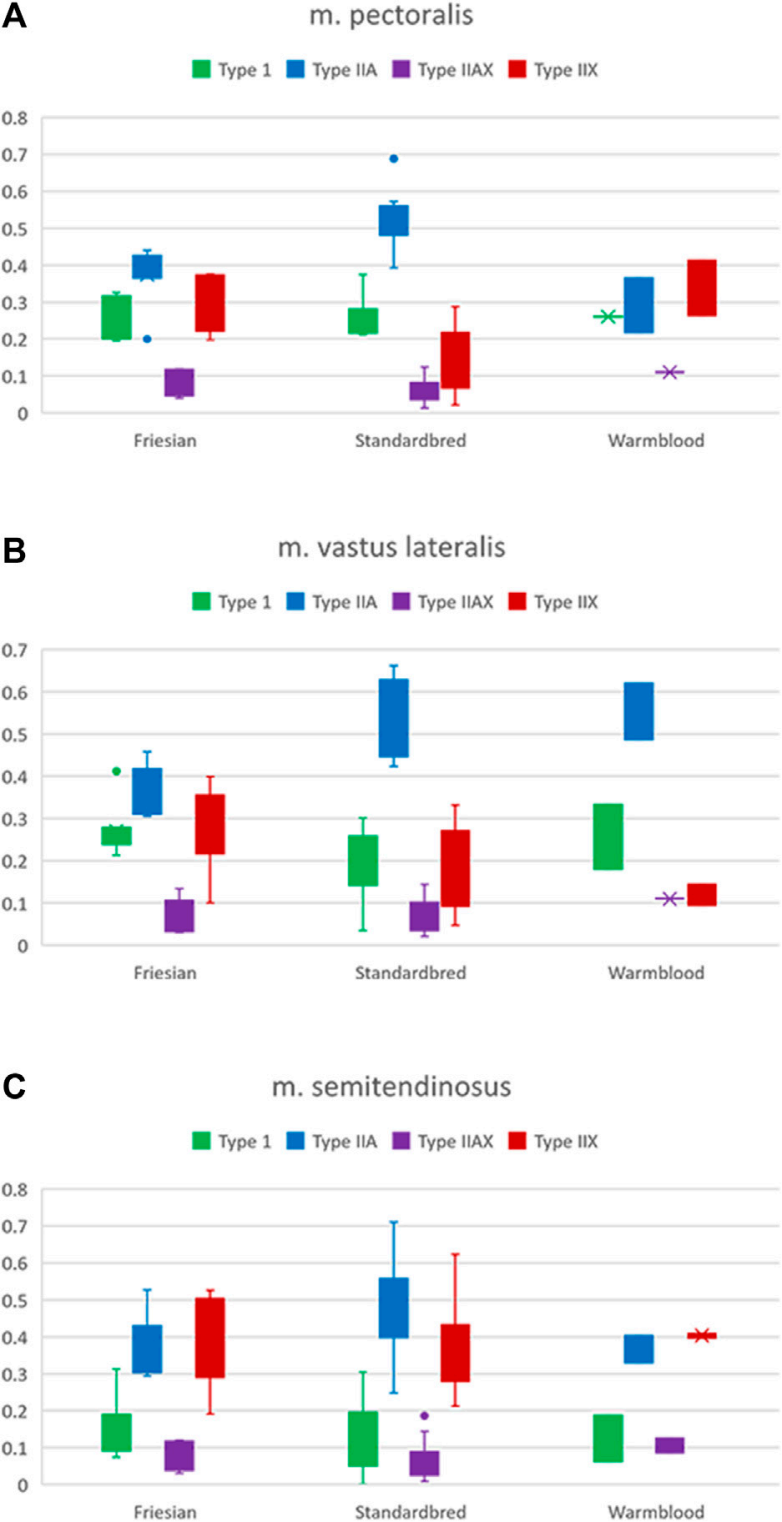
Boxplots of the percentages of type I (green), IIA (blue), IIAX (purple) and IIX (red) fibers in respectively Friesian, Standardbred and Warmblood horses for the **(A)** *m. pectoralis* (PM), **(B)** *m. vastus lateralis* (VL) and **(C)** *m. semitendinosus* (ST). For a view on significant differences see Table 2.

**FIGURE 5:**
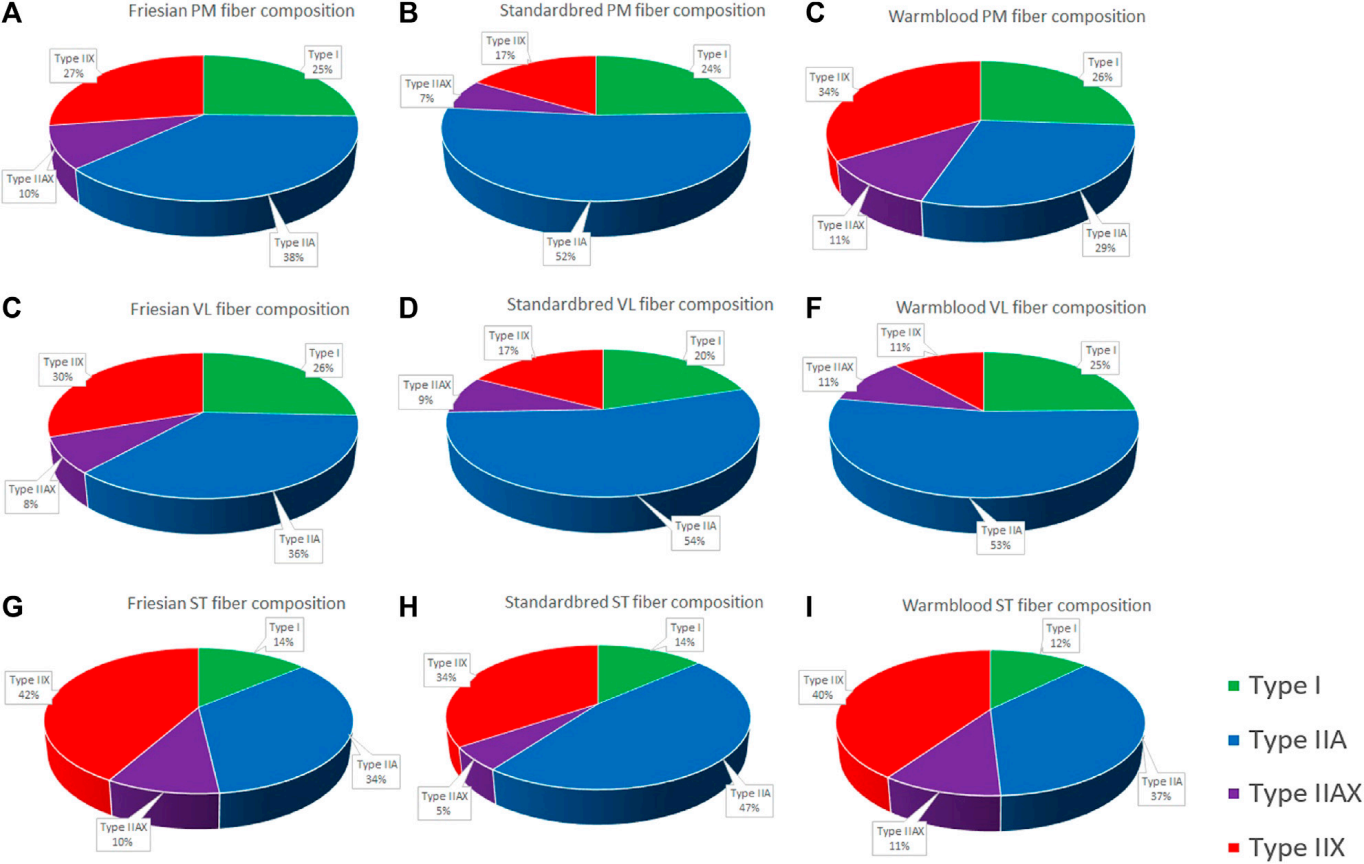
Pie charts of the fiber type composition of all studied muscle groups in all studied breeds expressed in percentage. Fiber type composition of the *m. pectoralis* (PM) in Friesian horses **(A)**; Standardbred horses **(B)**; and Warmblood horses **(C)**; Fiber type composition of the *m. vastus lateralis* (VL) in Friesian horses **(D)**; Standardbred horses **(E)**; and Warmblood horses **(F)**; Fiber type composition of the m. semitendinosus (ST) in Friesian horses **(G)**; Standardbred horses **(H)**; and Warmblood horses **(I)**.

In Standardbred horses, a significantly higher proportion of type IIA fibers was represented in both the PM (Standardbred vs. Friesian; *p* = 0.0003) and the VL muscle (Standardbred vs. Friesian; *p* = 0.0003). Friesian horses on their turn showed in their PM a significantly higher representation of type IIX fibers (Friesian vs. Standardbred; *p* = 0.0047) (Table 2).

### Breed differences in fiber specific cross-sectional area (fCSA)

No significant differences in fCSA in the PM (*p* = 0.346; *p* = 0.466; *p* = 0.073; *p* = 0.576), VL (*p* = 0.140; *p* = 0.087; *p* = 0.118; *p* = 0.363) and ST (*p* = 0.843; *p* = 0.509; *p* = 0.293; *p* = 0.335) could be detected across Friesian, Standardbred and Warmblood horses (Table 3).

**TABLE 3:** Median fiber cross-sectional area (fCSA) (µm^2^) and fCSA ranges for respectively type I, IIA, IIAX and IIX fibers for Friesian, Standardbred and Warmblood horses in the *m. pectoralis* (PM), *m. vastus lateralis* (VL) and *m. semitendinosus* (ST).

		PM			VL			ST		
		Friesian	Standardbred	Warmblood	Friesian	Standardbred	Warmblood	Friesian	Standardbred	Warmblood
Fiber cross-sectional area (µm^2^)	Type I	5664 (3320–7181)	4517.7 (2131.2–10103.7)	4564.5 (4098–5031)	2990 (2284–3925)	3108.2 (2314.3–4449)	4559.5 (4016–5103)	4207 (2470–8493)	5184.5 (2420.6–9917.8)	6154 (3868–8440)
	Type IIA	7806 (4917–13816)	5685.1 (3306.9–12516.5)	5829.5 (5443–6216)	5606 (4138–6994)	5670.1 (4096.9–8493)	8696 (8067–9326)	5743 (3825–8600)	5383.8 (3295.4–10689.8)	8235.5 (5640–10831)
	Type IIAX	8239 (4309–14601)	5921.1 (3590.5–25789.8)	6204.5 (5780–6629)	6021 (4727–8229)	6165.7 (4562.5–11306)	13566 (12632–14500)	5941 (4742–11342)	7077.2 (3701.8–13289.1)	10943 (6891–14995)
	Type IIX	8832 (6220–15602)	5684.4 (3407.5–15738.9)	6092.5 (6074–6111)	6195 (5039–8928)	5800.4 (4528.5–9988)	9708 (9182–10234)	8073 (6220–9687)	8489.2 (5484.8–12159.2)	11620.5 (9665–13576)

### Breed differences in mean fiber cross-sectional area (mfCSA)

**FIGURE 6:**
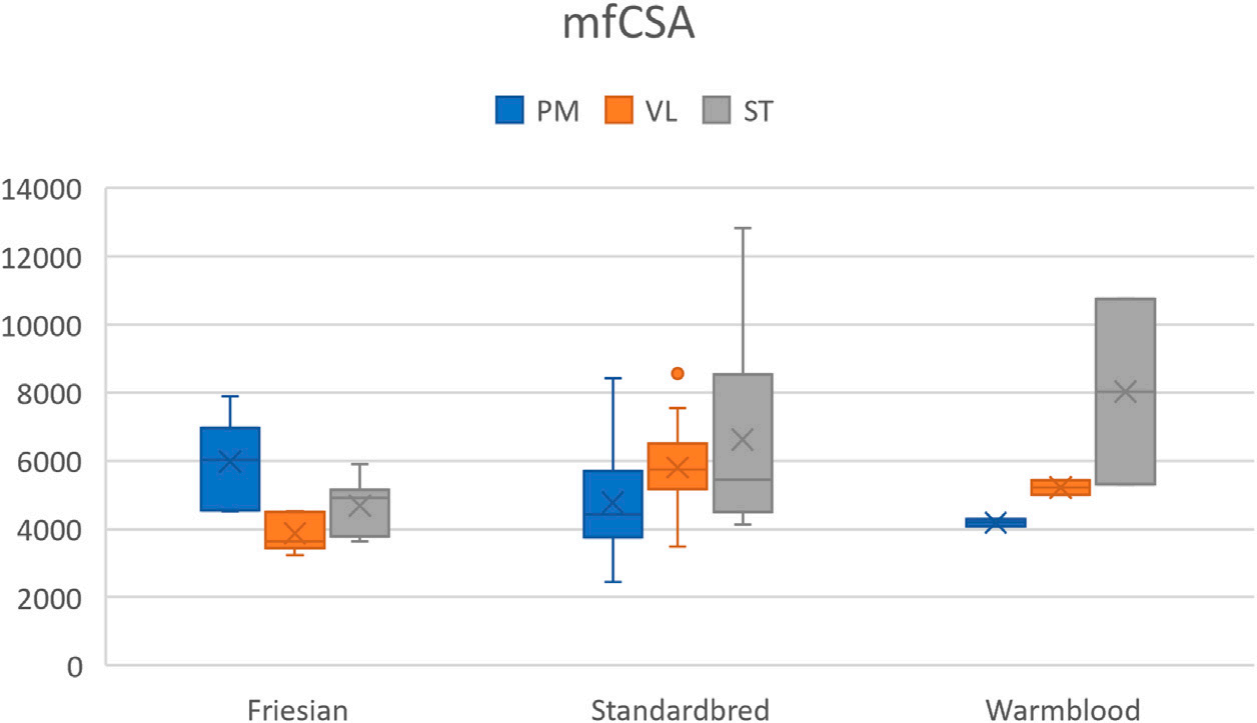
Boxplot of the mean fiber cross-sectional area (mfCSA) (µm^2^) in Friesian, Standardbred and Warmblood horses for the *m. pectoralis* (PM), *m. vastus lateralis* (VL) and *m. semitendinosus* (ST). For a view on significant differences see Table 4.

A significantly larger mfCSA (*p* = 0.0017) was seen in the VL of Standardbred horses [5740.3 (3487.1–8568.5) µm^2^] compared to Friesian horses [3643.7 (3225.8–4528.3) µm^2^] (Table 4). Though not statistically significant, Warmblood horses followed the same trend when compared to Standardbreds. The baseline profile of mfCSA for the three muscle groups in each horse breed is visualized in Figure 6.

**TABLE 4:** Median mean fiber cross-sectional area (mfCSA) (µm^2^) and mfCSA ranges of *m. pectoralis* (PM), *m. vastus lateralis* (VL) and *m. semitendinosus* (ST) in Friesian, Standardbred and Warmblood horses. Significant findings are highlighted in red.

	Mean fiber cross-sectional area (µm^2^)		
Breed	PM	VL	ST
Friesian	6024.1 (4524.9–7894.7)	3643.7 (3225.8–4528.3)	4909.5 (3644.3–5907.2)
Standardbred	4434.8 (2447.6–8426.9)	5740.3 (3487.1–8568.5)^*^	5451.7 (4122.3–12830.7)
Warmblood	4195.1 (4098.4–4291.8)	5214.3 (5000–5428.6)	8035.9 (5319.1–10752.7)

^*^Significantly higher value compared to Friesian horses.

### Breed differences in muscle baseline metabolic profile across PM and VL

Untargeted metabolomics was performed on the VL in all involved breeds, as well as on the PM of Friesian and Warmblood horses. Table 5 provides a summary of metabolites found significantly different in the baseline metabolic profile of the VL in Standardbreds *versus* Friesians on one hand, and Warmbloods *versus* Standardbreds, with their respective FD. A figure showing the average baseline concentration of metabolites for each of the sub pathways found in the VL of respectively Friesians, Standardbred and Warmblood horses can be found in the (Supplementary Figure S1). Supplementary Table S4 provides an overview of metabolites found significantly different in the baseline metabolic profile of the PM and the VL between Warmblood and Friesian horses with their respective FD.

**TABLE 5:** Summary of metabolites found significantly different in the baseline metabolic profile of the *m. vastus lateralis* (VL) in the comparisons Standardbred/Friesian and Warmblood/Standardbred with their respective fold difference (FD). Legend: dark red: values exceeding 2-FD; light red: values equal to 1 and lower than 2-FD; dark green: values below 0.5-FD; light green: values between 0.5 and 1-FD; blank cells correspond to metabolites that were not found significantly different or were only identified in one of the breeds. Left column: Standardbred vs. Friesian comparison: cells in red represent metabolites more active in Standardbred horses and cells in green represent metabolites more active in Friesian horses. Right column: Warmblood vs. Standardbred comparison: cells in red represent metabolites more active in Warmblood horses and cells in green represent metabolites more active in Standardbred horses.

Superpathway	Subpathway	Metabolite	Standardbred vs. Friesian (fold difference)	Warmblood vs. Standardbred (fold difference)
Lipid	Carnitine Metabolism	carnitine		1.1
	Diacylglycerol	diacylglycerol (16:1/18:2 [2], 16:0/18:3 [1])*	0.45	
	Fatty Acid Metabolism (Acyl Carnitine)	dihomo-linolenoylcarnitine (20:3n3 or 6)*	1.7	
		arachidonoylcarnitine (C20:4)	1.61	
		oleoylcarnitine (C18:1)	1.35	
		linoleoylcarnitine (C18:2)*	1.3	
		acetylcarnitine (C2)	0.87	
	Fatty Acid Synthesis	malonylcarnitine		1.75
	Glycerolipid Metabolism	glycerol 3-phosphate	0.58	
	Lysolipid	1-linoleoyl-GPC (18:2)		0.46
		1-oleoyl-GPC (18:1)		0.57
	Phospholipid Metabolism	glycerophosphoinositol*	1.99	
		1-stearoyl-2-linoleoyl-GPS (18:0/18:2)	0.78	
		1-stearoyl-2-arachidonoyl-GPI (18:0/20:4)	0.74	
		1-palmitoyl-2-oleoyl-GPC (16:0/18:1)		0.81
		1-stearoyl-2-linoleoyl-GPI (18:0/18:2)		0.74
		cytidine-5′-diphosphoethanolamine		1.55
	Sphingolipid Metabolism	N-stearoyl-sphingosine (d18:1/18:0)*	0.79	
		sphinganine	0.61	1.48
Amino Acid	Alanine and Aspartate Metabolism	asparagine		1.74
		N-acetylaspartate (NAA)		2.14
		N-methylalanine		2.16
	Glutamate Metabolism	glutamate		1.3
		glutamate, gamma-methyl ester		1.22
		glutamine		1.71
	Glutathione Metabolism	5-oxoproline		1.78
		glutathione, oxidized (GSSG)		1.35
	Glycine, Serine and Threonine Metabolism	betaine	1.23	0.74
		glycine		1.41
		threonine		1.3
	Leucine, Isoleucine and Valine Metabolism	3-hydroxybutyrylcarnitine (2)	0.57	0.56
		tiglylcarnitine (C5:1-DC)	0.41	
		alpha-hydroxyisocaproate	0.16	4.65
	Lysine Metabolism	2-aminoadipate		2.43
		glutarylcarnitine (C5-DC)		2.06
		lysine		1.84
		N6-acetyllysine		1.5
		pipecolate		0.48
	Phenylalanine and Tyrosine Metabolism	phenyllactate (PLA)	0.23	4.97
	Tryptophan Metabolism	indolelactate	0.26	2.7
	Urea cycle; Arginine and Proline Metabolism	N-delta-acetylornithine		0.35
		N-methylproline		0.5
Carbohydrate	Advanced Glycation End-product	N6-carboxymethyllysine		1.72
	Aminosugar Metabolism	N-acetylglucosaminylasparagine		1.43
	Glycolysis, Gluconeogenesis, and Pyruvate Metabolism	lactate	1.58	
		3-phosphoglycerate	0.18	
		phosphoenolpyruvate (PEP)	0.08	0.55
	Pentose Phosphate Pathway	ribose 1-phosphate		0.57
Energy	Oxidative Phosphorylation	acetylphosphate	0.57	
	TCA Cycle	2-methylcitrate/homocitrate	1.94	
		aconitate [cis or trans]	0.31	3.58
Nucleotide	Purine Metabolism, (Hypo)Xanthine/Inosine containing	inosine 5′-monophosphate (IMP)	0.72	
	Purine Metabolism, Adenine containing	adenosine 3′,5′-diphosphate	1.59	0.53
		adenosine 5′-monophosphate (AMP)	0.37	
		adenosine		0.4
	Purine Metabolism, Guanine containing	guanosine 5′- monophosphate (5′-GMP)	0.4	0.33
		guanosine		0.46
	Pyrimidine Metabolism, Cytidine containing	cytidine 5′-monophosphate (5′-CMP)	0.67	
		cytidine	0.45	
	Pyrimidine Metabolism, Uracil containing	uridine	0.81	
		3-ureidopropionate		1.77
		uridine 5′-monophosphate (UMP)		0.16
Peptide	Gamma-glutamyl Amino Acid	gamma-glutamylhistidine	0.72	
		gamma-glutamylvaline	0.53	
Xenobiotics		4-ethylphenylsulfate	0.27	1.63
		4-methylcatechol sulfate		0.3
		sulfate*	2.66	
		quinate	2.9	
		N-glycolylneuraminate	1.71	
		methyl glucopyranoside (alpha + beta)		2.12

Across breeds the VL showed a wider array of differences between energy super pathways when compared to the PM. The lipid and nucleotide super pathways were significantly more active in Friesian horses when compared to Standardbreds, especially short and medium-chain acylcarnitines, however, in conjunction with high levels of long-chain fatty acids and polyunsaturated fatty acids (PUFAs) compared to Warmblood horses. No differences were seen in short-chain fatty acids (SCFA). Standardbred horses were very high in xenobiotic pathways and within the lipid super pathway, long and very long-chain acylcarnitines were clearly much more abundant when compared to Friesian horses. The amino acid metabolism showed similar activity across both breeds, except for the BCAA and AAA sub pathways which were significantly more active in Friesian horses. The carbohydrate, amino acid and nucleotide super pathways and carnitine metabolism showed significantly higher activity in Warmblood when compared to Standardbred horses. A figure showing the average baseline concentration of metabolites for each of the sub pathways found in the m. pectoralis (PM) of Friesian and Warmblood horses can be found in the Supplementary Figure S2.

### Standardbred vs. Friesian

The lipid and nucleotide super pathways (Table 5; Supplementary Figure S1) showed significantly higher activity in Friesian horses when compared to Standardbred horses. Standardbred horses on their turn showed significant higher activity of the xenobiotic pathways and within the lipid super pathway, acylcarnitines, more specifically long and very-long chain acylcarnitines, were significantly more active when compared to Friesian horses. The FD, fold difference, between breeds for each metabolite can be found in Table 5.

When focusing separately on each of the super pathways, following important breed differences could be discerned:

In lipid metabolism, Friesian horses generally exhibited higher activity, except for the acylcarnitine sub pathway, which was more active in Standardbred horses (1.30–1.70 FD). This disparity extended across various lipid sub pathways like diacylglycerol (0.45 FD), glycerolipid (0.58 FD), sphingolipid (0.79 and 0.61 FD), and phospholipid (0.74–0.78 FD) sub pathways, all displaying higher activity in Friesian horses, except for glycerophosphoinositol (1.99 FD), notably higher in Standardbreds.

The carbohydrate sub pathways followed a less straight forward trend across both breeds. For example, lactate (1.58 FD) and methylcitrate/homocitrate (1.94 FD) were clearly more abundant in Standardbred horses, as opposed to metabolites like 3-phosphoglycerate (0.18 FD) and phosphoenolpyruvate, which were less abundant in Standardbred horses compared to Friesians. Other glycolysis, gluconeogenesis and pyruvate cycle metabolites such as 3-phosphoglycerate and phosphoenolpyruvate were less abundant in Standardbred horses when compared to Friesian horses. Likewise, several metabolites of the TCA cycle and oxidative phosphorylation (OXPHOS) system such as acetylphosphate (0.57 FD) and aconitate (0.31 FD), were less active in Standardbred horses when compared to Friesian horses.

Amino acid metabolism, showed consistent activity between the two breeds, except for certain subpathways like BCAA (0.31–0.74 FD), AAA (0.23–0.26), which were notably less active in Standardbred horses, while betaine (1.23 FD) displayed higher activity in this breed (Table 5, left column).

The nucleotide super pathway was significantly less active in Standardbred horses when compared to Friesian horses. Adenosine 3′,5′-diphosphate (1.59 FD), from the adenine containing purine metabolic sub pathway, was the only metabolite showing more activity in Standardbred horses compared to Friesian horses.

Xenobiotic sub pathways were significantly more active in Standardbred horses when compared to Friesian horses, specifically the metabolites sulfate (2.66 FD), quinate (2.9 FD) and N-glycolylneuraminate (1.71 FD), except for one metabolite, 4-ethylphenylsulfate from the benzoate metabolism (0.27 FD).

### Warmblood vs. Standardbred

At the level of the super pathways, both Warmblood and Standardbred showed equal activity at the level of the lipid metabolism, with the exception of a more pronounced activity in Warmblood horses of the carnitine metabolism (1.10 FD), fatty acid synthesis (1.75 FD) and cytidine-5′-diphosphoethanolamine (1.55 FD) and sphinganine (1.48 FD) (Table 5, right column). Also, the xenobiotic super pathway showed similar activity between both breeds except for methyl glucopyranoside (alpha and beta) (2.12 FD) and 4-ethylphenylsulfate (1.63 FD) being more active in Warmblood horses when compared to Standardbred horses. All other super pathways (carbohydrate/energy, amino acid and nucleotide) showed significantly higher activity in Warmblood when compared to Standardbred horses.

In the carbohydrate/energy super pathway especially advanced glycation end-products (1.72 FD), amino sugar metabolites (1.43 FD) and aconitate (3.58 FD) were significantly more abundant in Warmblood when compared to Standardbred horses.

In the amino acid super pathway most metabolites were significantly higher in Warmblood when compared to Standardbred horses. Important highlights with that respect are N-acetylaspartate (2.14 FD) and N-methylalanine (2.16 FD), alpha-hydroxyisocaproate (4.65 FD), 2-aminoadipate (2.43 FD) and glutarylcarnitine (2.06 FD), and finally phenylalanine and tyrosine (4.97 FD) and tryptophan (2.70 FD). In contrast, certain metabolites including betaine (0.74 FD), 3-hydroxybutyrylcarnitine (0.56 FD)and pipecolate (0.48 FD) exhibited higher activity in Standardbred horses. All amino acids from the urea cycle, specifically arginine and proline metabolism, were also more active (0.35–0.5 FD) in Standardbred horses.

The nucleotide super pathway was less active in Warmblood horses when compared to Standardbred horses, except for 3-ureidopropionate (1.77 FD) from the pyrimidine metabolism.

### Warmblood vs. Friesian

All energy super pathways showed similar trends in their breed comparisons, with the exception of the xenobiotic and the nucleotide super pathways, which showed only clear breed differences in the locomotor muscle (VL) as opposed to the PM which showed no breed differences. The results show differential activity across breeds and muscles (Supplementary Table S4, left (PM) and right column (VL); Supplementary Figures S1, S2).

In the lipid metabolism a clear breed-based distinction was visible across both muscle groups at the level of the acylcarnitine metabolism: both short- and medium-chain metabolites (Short chain (C2-C5; 0.73–0.86 FD PM; 0.84–0.89 FD VL); Medium chain (C6-C13; 0.54–0.86 FD PM; 0.75–0.93 FD VL) are all more active in Friesians when compared to Warmblood horses, whereas the long-chain (C14-C21; 1.01–1.38 FD PM; 1.17–2.09 FD VL) and very long-chain (≥C22; 1.06–1.39 FD PM; 1.47–2.48 FD VL) acylcarnitines are higher in Warmblood horses. Moreover, Warmbloods displayed significantly more active carnitine metabolism compared to Friesians, particularly in the VL, showcasing notably high levels of certain metabolites like arachidonoylcarnitine (2.01 FD), adrenoylcarnitine (2.48 FD), dihomo-linolenoylcarnitine (2.09 FD) and docosapentaenoylcarnitne (2.22 FD). Long-chain fatty acid andpolyunsaturated fatty acids were more abundant in Friesian horses when compared to Warmblood horses, except for valerate, (1.2 FD (2.24 FD) which was notably higher in Warmblood horses, particularly in the VL.

In the carbohydrate/energy sub pathways, several pivotal metabolites in the PM were more abundant in Warmblood horses when compared to Friesians, such as succinylcarnitine (1.84 FD), fumarate (1.2 FD), malate (1.18 FD) and 2-methylcitrate/homocitrate (1.84 FD), though again, this was even more pronounced in the VL for which especially succinylcarnitine and 2-methylcitrate/homocitrate (2.39 FD) are very active in Warmblood horses.

With respect to the amino acid sub pathways, most showed similar breed trends across both muscle groups except for AAA and BCAA metabolism, which appeared more prevalent in Friesians. Specific metabolites, like 3-hydroxyphenylacetatoylcarnitine (6.98 FD PM; 4.37 FD VL), cis-urocanate (4.58 FD PM; 2.85 FD VL), pyroglutamine (2.95 FD PM; 2.23 FD VL), S-methylcysteine sulfoxide (2.41 FD PM; 2.44 FD VL), taurocyamine (3.22 FD PM; 2.73 FD VL), glycylleucine (2.43 FD PM; 2.04 FD VL) and valylleucine (2.56 FD PM; 2.03 FD VL) were highly active in Warmbloods, whereas N-delta-acetylornithine (0.36 FD PM; 0.4 FD VL), 4-hydroxy-nonenal-glutathione (0.11 FD PM; 0.22 FD VL) and pipecolate (0.47 FD PM; 0.48 FD VL) were particularly less abundant in Warmblood horses compared to Friesian horses.

Only in the VL muscle, all nucleotide sub pathways observed, except the thymine containing pyrimidine 3-aminoisobutyrate (1.58 FD), were less active in Warmblood horses when compared to Friesian horses.

While the nucleotide and xenobiotic super pathways showed no discernible breed differences in PM, in VL muscle, Warmblood horses exhibited increased activity in the xenobiotic super pathway, notably featuring metabolites like benzoylcarnitine (1.96 FD), N-glycoylneuraminate (2.65 FD), erythritol (5.82 FD) and methyl glucopyranoside (2.70 FD), while 4-ethylphenylsulfate (0.44 FD), 4-methylcatechol sulfate (0.34 FD), 4-acetylphenol sulfate (0.28 FD) and umbelliferone sulfate (0.07 FD) were much less abundant in Warmblood horses when compared to Friesian horses.

### Associations between the metabolic profile and other physio-morphological features

No significant associations could be found between the metabolic blueprints and the assessed physio-morphological parameters such as fiber type composition, mCSA and mfCSA. (Supplementary Tables S1–S3).

## Discussion

Linking muscular histological parameters to metabolic output is crucial for better understanding the functional aspects of muscle tissue. It is essential to understand adaptation to training, to optimize disease and recovery monitoring and to optimize nutritional interventions. To the best of our knowledge, this is the first study to baseline morpho-physiological parameters in three different horse breeds across three muscle groups combining histomorphology with untargeted metabolomics. Although horses and humans differ greatly from each other, even in terms of diet and GI physiology, it is to be expected that each species physiologically has a certain muscular main-frame metabolism, as well as a certain set of fallback scenarios. What represents an additional fallback scenario or a metabolic pathway that is active in the background in one species may be the main pathway for another species. Extrapolating results from human training studies to horses and *vice versa* should therefore be done with caution. However, the comparative approach can teach us a lot here. Horse breeds are the result of thousands of years of selection to excel in a particular discipline. They are often controlled by closed studbooks, which makes them genetically much more uniform than, say, a group of human athletes that excel in, for example, sprint capacity. This study does not intend to suggest that the combination of morphophysiological factors and metabolic output found in horse breeds that for example are strong in power output (Friesian horse), can be extrapolated one on one to humans that excel in power sports, but these study results are important to also apply to humans, to broaden human physiological metabolic insights. What is a crucial pathway in a horse, for example, can represent 10% of the total metabolic output for a human athlete, which is already crucial knowledge in terms of training and nutritional interventions.

### The added value of comparing different archetypical breeds and including posture *versus* locomotion muscle groups

In the current study, three importantly different horse breeds were compared: Friesian *versus* Warmblood *versus* Standardbred horses, all genetically selected towards an ultimate specific performance skill. The strict offspring control that is being exercised over the different studbooks representing these different breeds ensures that the study populations involved are quite uniform. The breed differences found can therefore be better linked to the genetic and exercise archetype and thus the metabolic fingerprint being identified. With that respect, Friesian horses can be viewed as the “bodybuilders” of the equine world, Standardbred horses as sprinters that perform their exercise in a predominant aerobic fashion and Warmblood horses can be viewed as the most versatile breed, engaging in all kinds of different types of competition such as dressage, show jumping and eventing. Therefore, from all involved Studbooks, the Warmblood Studbook is the most versatile.

Involving three different muscle groups also adds to creating a more complete view on how locomotor *versus* posture predominant muscle groups can fulfill their specific function. Muscles can be broadly classified into two types based on their function and metabolic characteristics: posture muscles and locomotion muscles. These classifications are associated with the primary functions these muscles serve in the body. Posture muscles are primarily responsible for maintaining body posture and stability. They work to counteract the force of gravity, allowing an individual to stand or sit upright. Locomotion muscles are involved in movement and generating forces required for activities such as walking, running, jumping, and other dynamic actions. Up until now, the distinction between locomotor and postural muscles is quite arbitrary in the horse, though also with that respect, quite some research has already been performed (Payne et al., 2005a; Payne et al., 2005b; de Meeûs d’Argenteuil et al., 2021a). Understanding differences between posture and locomotion muscles is essential for designing effective training programs, rehabilitation strategies, and optimizing overall muscular function based on specific functional requirements, whether it be maintaining posture or engaging in locomotion activities. With that respect, apparently, the ST muscle fulfills a similar locomotory role across all equine breeds involved in this study. Indeed, it is the least breed discriminative muscle (Figures 3, 4), which is an interesting finding that needs to be further scrutinized in follow-up studies. Up until now, the GM is mostly used in muscular and training studies across species and breeds (Snow and Guy, 1980; Valberg, 1987; López-Rivero et al., 1989; López-Rivero et al., 1991; Rivero et al., 1995; Islas et al., 1997; van Ginneken et al., 2006; Lindner et al., 2013; Valberg et al., 2022). The ST, on the other hand, has rarely been used to that purpose although this muscle allows for easy and repeated sampling without complications. Its anatomical location allows for optimal drainage of the biopsy wound and for strategic sampling, keeping in mind gravity and thus avoiding “contamination” of the next sampling sites caused by drainage fluids with the potential of activating inflammatory pathways that can skew study results (Vissing et al., 2005; Thienen et al., 2014; de Meeûs d’Argenteuil et al., 2021b; de Meeûs d’Argenteuil et al., 2021a). The VL showed a wider array of differences across breeds for the studied physiological parameters when compared to the PM. In the past, we have shown that in Friesian horses the m. pectoralis does cover a locomotory function, probably to dynamize the high knee action, which is a gait-specific feature for this breed (de Meeûs d’Argenteuil et al., 2021a).

With respect to the three involved morphophysiological parameters in the current study (muscle fiber typing, fCSA, mfCSA), the fiber specific CSA was the least discriminative between breeds, suggesting that the corollary of type I fibers being small and highly capillarized allowing for swift muscle fuel supply, stands across breeds. The same goes for type IIX fibers, typically large to allow for storage of reserve fuels, which are rapidly consumed by this type of muscle fiber during explosive bouts of exercise. When looking across species, this paradigm does not remain valid. Indeed, in humans, type IIX fibers are smaller than type IIA fibers (Bloemberg and Quadrilatero, 2012; Jeon et al., 2019). The aforementioned could illustrate that for example type IIX fibers do not rely on the same fast metabolic machinery across species. Type IIX fibers represent the capacity to sustain explosive bouts of exercise, predominantly fuelled by predominant anaerobic metabolism. Since the “*ad hoc*” need to supply energy demands, it is feasible that differences in dietary habits across the aforementioned species, explain the reported differences with respect to fCSA of type IIX seen across species. Interestingly, type I fibers, however, do seem to follow the same paradigm across species and breeds. Rivero and Diz (1992) for example reported similar results for the GM upon comparing three different horse breeds: Andalusian, Thoroughbred and Arab horses (Rivero and Diz, 1992). Also, in a study performed in ducks by Li and co-workers, no significant differences were found in the fCSA between the breeds examined (Li et al., 2016). Similarly, in a study by van Boom et al. (2023), the fCSA in two different muscles of 16 dog breeds showed no significant differences. Overall, this suggests that the fCSA is not discriminative between breeds of the same species and that species mainly differ in the fCSA of non type I fibers.

The mfCSA on the contrary, representing the mean CSA across all muscle fiber types, clearly shows significant breed differences. Indeed, Standardbred horses showed a significantly larger mfCSA (Table 4) and were richer in type IIA fibers, both in the PM and VL muscle compared to Friesian horses. Warmblood horses demonstrated the same trend of larger mfCSA compared to Standardbred horses, although results did not attain significance.

When focusing on muscle fiber typology and comparing between breeds in the current study, the representation of type I fibers showed most equality across studied muscles and breeds (Table 2; Figures 3, 4). By contrast, type IIA and type IIX fibers were most discriminative between breeds. It is more than likely that this is due to the fact that precisely these muscle fiber types have to realize a much greater versatility of exercise per breed when compared to type I fibers, the latter of which are most represented in posture predominant muscle groups. Standardbred horses showed the highest representation of type IIA fibers in both the PM and VL muscle when compared to Friesians and Warmblood horses, which matches with the type of exercise for which they are bred. Indeed, type IIA fibers are known as the predominant fiber type in horses (Snow and Guy, 1980; Gunn, 1987; Rivero and Hill, 2016) and it seems that Standardbred horses represent an archetypical model in that respect. While the fiber type composition in humans shows high levels of both type I and IIA fibers (Jeon et al., 2019), training can increase the ratio of type II fibers. Unfortunately, most currently available studies have focused on I and IIA fibers exclusively (Metaxas et al., 2019; Gejl et al., 2021). Friesian horses have a very high representation of type IIX fibers in their PM, compared to other breeds. Our research team has shown in the past that the PM of Friesian horses covers a more pronounced locomotory function compared to other breeds, possibly because of their involvement in high knee action in different gaits. Overall, Warmblood and Standardbred horses were quite comparable at the level of fiber type composition across sampled muscles (Figure 5).

The fiber type composition data obtained in this study are in accordance with previous studies on untrained Standardbred and Dutch Warmblood horses (Essén et al., 1980; Taylor and Brassard, 1981; Karlstrom et al., 1994; Grotmol et al., 2002; van Ginneken et al., 2006). The type IIA fiber representation in both PM and VL in Standardbred horses compared to Friesian horses found in the present study is coherent with the predominant aerobic sprinting activities for which Standardbred horses are bred (Taylor and Brassard, 1981; Rivero et al., 1993). This is comparable with humans, especially men, in which type IIA representation in the VL is also high (Bloemberg and Quadrilatero, 2012; Jeon et al., 2019).

### Zooming in on metabolic steady-state condition differences across archetype equine breeds being conceptualized for top performance in different sports disciplines

The results in the current study demonstrate that there are important metabolic differences between equine breeds, which need further attention in future studies and when formulating training and dietary management protocols. Though all three involved equine breeds show important differences, Standardbred horses and Warmblood horses seem to be more “alike” when compared to Friesian horses (Figure 5). Also of interest is the fact that these metabolic differences are expressed by both PM and VL across breeds, indicating that, despite differences in physiological role between muscle groups, these specific metabolic differences stand across breeds, though more thoroughly expressed in locomotor muscles.

**FIGURE 7:**
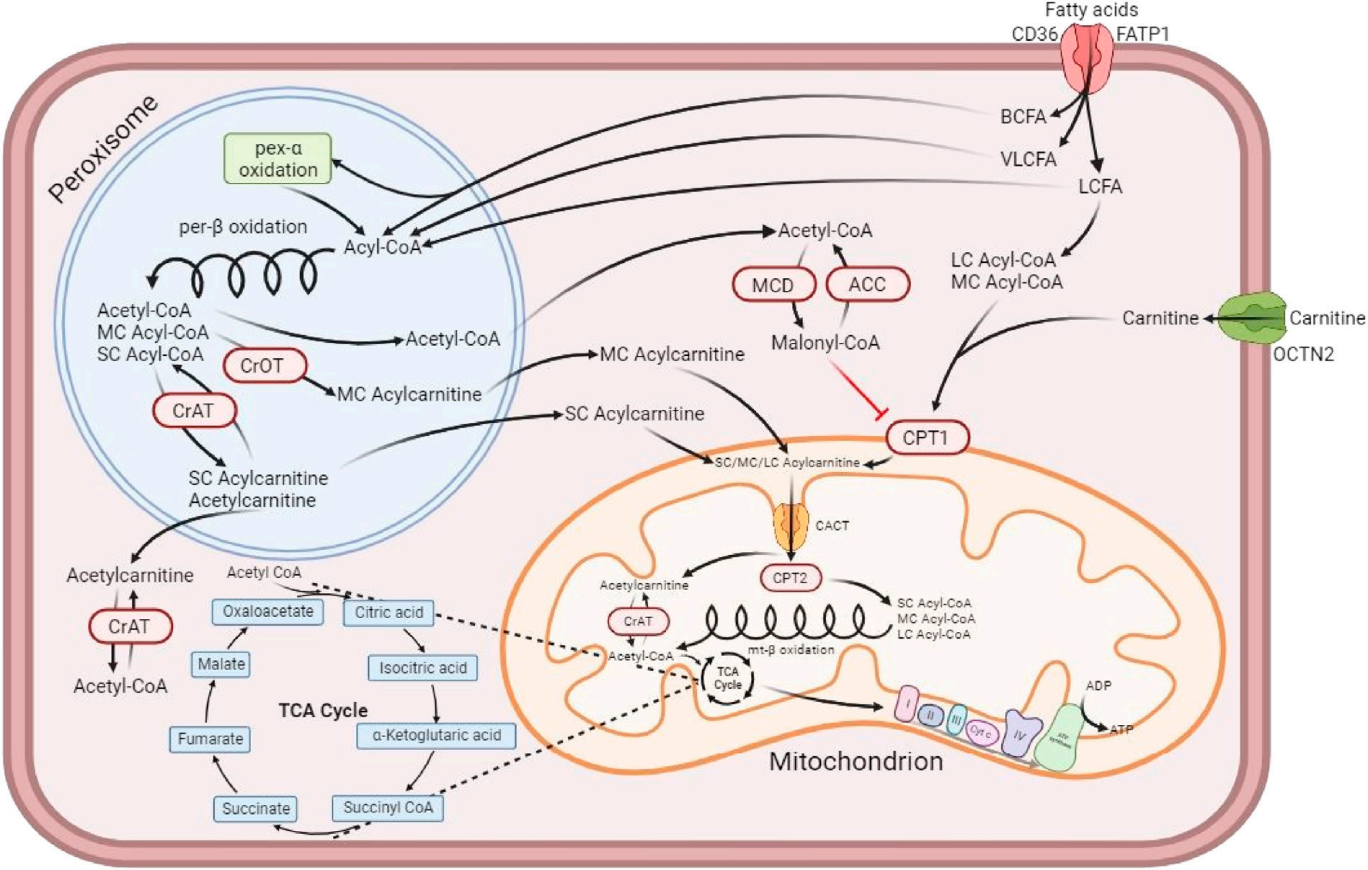
Muscle cell depicting the formation of acylcarnitines, their transport into the mitochondrion and their participation in the production of ATP. Adapted from Dambrova et al. (2022) and created with BioRender. Fatty acids access the cell via the fatty acid translocase CD36 and the fatty acid transporter protein 1 (FATP1) (Hao et al., 2020; Dambrova et al., 2022). Branched-chain fatty acids (BCFAs) and long and very long-chain fatty acids (LCFAs and VLCFAs) enter the peroxisome, where each of them undergo the peroxisomal β-oxidation resulting in simpler medium and short-chain fatty acids (MCFAs and SCFAs). Some of the short-chain acylcarnitines (SCACs), such as butyrylcarnitine and propionylcarnitine, come mainly from the catabolism of branched-chain amino acids (BCAA), while others like acetylcarnitine are only slightly affected by that metabolic process (Halama et al., 2016). While some SCACs are produced in the peroxisome, the majority of SCACs are produced by the reaction catalyzed in the cytosol by a carnitine acyltransferase (CrAT) enzyme that binds L-carnitine to a short-chain fatty acid, for example acetyl-CoA, which is then transformed into an acylcarnitine, in this case acetycarnitine (Adeva-Andany et al., 2017; Dambrova et al., 2022). Short-chain fatty acids (SCFAs) are synthesized in the colon by the microbiota present there, with acetate, propionate and butyrate being the most released through fermenting fiber and resistant starches (Tan et al., 2014). After binding to carnitine, SCFAs can enter the outer membrane of the mitochondria and go through the inner membrane via the carnitine-acylcarnitine translocase (CACT); once inside, they can participate as substrate for the β oxidation cycle, after being separated from carnitine by carnitine palmitoyltransferase 2 (CPT2) (Engelking, 2015). Medium-chain acylcarnitines (MCACs), on the other hand, are produced in the peroxisome via the carnitine octanoyltransferase (CrOT), which can partially contribute to MCAC production from the metabolism of medium- and long-chain fatty acids (MCFAs and LCFAs) and the breakdown of very long-chain acylcarnitines (VLCACs) (Adeva-Andany et al., 2017; Dambrova et al., 2022). Despite the difference in their synthesis compared to SCACs, the function of MCACs is also to feed into the mitochondrial β-oxidation by following the same process (Engelking, 2015; Dambrova et al., 2022). Long-chain fatty acids, as opposed to VLCFAs, can also enter the mitochondria to participate in the β oxidation. They must be bound first to carnitine via the carnitine palmitoyltransferase 1 (CPT1) to access the intermembrane space as long-chain acylcarnitines (LCACs) and then go through CACT to pass the inner membrane of the mitochondria into the matrix, where they lose their carnitine thanks to CPT2. Only then they can participate in the mitochondrial β-oxidation (Dambrova et al., 2022). Additionally, acetyl-CoA accumulation in the cytosol can give rise to the production of malonyl-CoA via the malonyl-CoA decarboxylase (MCD), which is reversible by acetyl-CoA carboxylase (ACC). The accumulation of malonyl-CoA can inhibit the production of LCACs from LCFAs, impairing the β-oxidation (Dambrova et al., 2022). The organic cation transporter novel family member 2 (OCTN2) is the high affinity protein transporter that allows the refill of the carnitine pool in the cytosol. Another Friesian specific feature was the high activity of almost all nucleotide sub pathways in the VL compared to the other two breeds. Fast-twitch muscle fibers are known to display a much higher rate of nucleotide synthesis, especially adenine, when compared to slow-twitch fibers, due to the high speed at which fast-twitch processes take place (Tullson and Terjung, 1991). The fact that both type IIX and type IIA fibers are vastly represented in Friesian horses, could serve as an explanation for this finding.

An interesting finding was the fact that the lipid and nucleotide super pathways were significantly more active in Friesian horses when compared to Standardbreds, especially short and medium-chain acylcarnitines. Interestingly, it is known that some of the short-chain acylcarnitines (SCACs), such as butyrylcarnitine and propionylcarnitine, mainly originate from the catabolism of branched-chain amino acids (BCAA), which were clearly more active in the Friesian breed (Halama et al., 2016). Further research is needed with that respect, but it is clear that this is a Friesian-specific feature. Another source of short-chain fatty acid (SCFAs) production is the gut microbiome, which is an important metabolite source in horses (Tan et al., 2014; Kauter et al., 2019; Mach et al., 2021). After binding to carnitine, SCFAs can enter the mitochondria through the carnitine shuttle, for further processing by the β oxidation pathway (Engelking, 2015) (Figure 7). Finally, the breakdown of medium-, long- and very long-chain fatty acids (respectively MCFAs, LCFAs and VLCFAs) can contribute to the origin of short and medium chain acylcarnitines (Adeva-Andany et al., 2017; Dambrova et al., 2022). An overview of the processing of SCFAs, MCFAs, and LCFAs and associated formation of acylcarnitines can be found in Figure 7. Carnitine itself is one of the metabolites that appears most active in Warmblood horses throughout the study (Figure 5). It is known that carnitine is essential for the shuttling of fatty acids from the cytosol into the mitochondria (Buechler and Lowenstein, 1990; Hoppel, 2003) (Figure 7). Finally, in an equine study comparing Guanzhong horses, which have athletic tall bodies, and Ningqiang pony horses, which have an agile but strong body that allows them to transport heavy goods in the mountains, the SCAC propionylcarnitine appeared in higher concentrations in the GM of the pony draft horses, which was attributed to the differences in muscle development between these two breeds (Meng et al., 2023). Interestingly, both in healthy untrained humans and endurance horses, such as Arabian and Standardbred horses, SCACs are the most abundant carnitine metabolites in blood, particularly acetylcarnitine (Bene et al., 2005; Westermann et al., 2008; Adams et al., 2009; van der Kolk et al., 2020). No blood sampling was performed in the current study. It was decided not to do so, since blood as matrix performs cross-talk with many body compartments, including GI tract, liver, muscle compartment, lungs, etc., and hence represents a complex metabolic mirror which not only illustrates what happens inside the muscular compartment.

Outstanding metabolic features for Standardbred horses were the high xenobiotic activity and within the lipid super pathway, the prominent representation of long and very long-chain acylcarnitines. Amongst these xenobiotics, was sulfate, which is the key metabolite for the formation of adenosine 3′,5′-diphosphate and is produced during cysteine and methionine metabolism (Kanehisa, 2019; Kanehisa et al., 2021). Quinate was also a metabolite noticeably active in Standardbred horses compared to Friesian horses. Quinate is a compound that is part of the shikimate pathway, a metabolic pathway found in plants, bacteria, and fungi. The shikimate pathway is responsible for the biosynthesis of aromatic amino acids, such as phenylalanine, tyrosine, and tryptophan. In mammals, including humans, the shikimate pathway is absent. Therefore, quinate itself is not directly involved in mammalian metabolism in the same way it is in plants. Quinic acid, a derivative of quinate, can be produced by the gut microbiome through microbial metabolism. Throughout the study, the same concentrate feed was used, however, roughage sources did differ. Quinic acid, its derivative, can undergo metabolism in the liver. Studies have shown that quinic acid can be converted to other metabolites that may have bioactive properties, for example antioxidant properties, potential anti-inflammatory effects, and its role in supporting gut health (Nam et al., 2018; Choi et al., 2021; Benali et al., 2022). The variability in its presence in the studied horse breeds may be due to differential use of roughage sources and/or due to differences in gut microbiome metabolic output. The benzoate metabolic sub pathway appeared less active in the VL of both Warmblood and Standardbred horses compared to Friesian horses, while other xenobiotic sub pathways were more active in those two breeds compared to Friesian horses. Interestingly, the degradation of benzoate is related to metabolites that end up feeding the TCA cycle (Wishart et al., 2018).

The other standardbred-specific metabolic feature was the high representation of long-chain (LCACs) and very long-chain acylcarnitines (VLCACs). Those are generated through L-carnitine esterification of long and very long-chain fatty acids that originate either from the diet or are generated by *de novo* lipogenesis and subsequent mobilization of fatty acids from the fat depots (Dambrova et al., 2022). Very long-chain fatty acids (VLCFAs) are too long to be part of the β-oxidation. Consequently, VLCFAs are metabolized in peroxisomes, where enzymes cleave them into shorter fragments that form short- and medium-chain acylcarnitines (Ferdinandusse et al., 2004; Dambrova et al., 2022) (Figure 7). In a human nutritional study, the plasma metabolome of groups with different diets (i.e.: vegan, vegetarian *versus* omnivore) showed that there was a markedly lower abundance in long-chain acylcarnitines in the vegan group (Miles et al., 2022). Horses have a pure vegan diet and Standardbreds as well as Warmblood horses displayed increased presence of long and very long chain acylcarnitines, i.e. opposite to the human response. Friesian horses on the other hand displayed increased short and medium chain acyl carnitines. Clearly, more research is needed to understand those differences; in that respect it would be interesting to compare fatty acid and acylcarnitine profiles in resting and acute exercise muscle biopsies and microbiome metabolic output. In an equine study that compared baseline amino acid and acylcarnitine concentrations in peripheral blood of Quarter horses and American Miniature horses, the authors found a breed effect on the concentration of acylcarnitines of all lengths, which was attributed to the variation in daily physical activity, individual genetics or environmental factors since the horses had been fed the same diet throughout the study, as was the case in the current study (Rodríguez-Sánchez et al., 2015). Further work is necessary to improve our understanding of muscular acylcarnitine dynamics upon physical activity relative to the resting condition (Assenza et al., 2012; Boemer et al., 2017).

As mentioned previously, when comparing the lipid metabolism across breeds it is important to keep in mind that in conjunction with a high expression of short and medium-chain acylcarnitines in Friesian horses, there was a significantly increased presence of long-chain fatty acids, whereas short-chain fatty acids were equally abundant across breeds. Since acylcarnitine formation occurs further downstream from fatty acid mobilization, this indicates that Friesian horses are less adept at the swift processing of long and very long chain fatty acids compared to Standardbred and Warmblood horses, which is logic since these fatty acids are used under endurance conditions and Friesian horses are not bred for that purpose (Nelson and Cox, 2017a). More research is needed in that respect, but the observation could be of importance when feeding Friesian horses. The more pronounced anaerobic fast-twitch muscle fiber profile seen in Friesian horses could partly function as an explanation for this finding. The higher percentage of type IIA fibers in the VL of Standardbred horses compared to Friesian horses has to be considered as well. Although this difference is not seen in an association between fiber type composition and baseline metabolic profile in any of the breeds in the current study, a more oxidative profile for both Standardbred and Warmblood horses does make sense. Also important to notice is the fact that the processing of long and very long-chain acylcarnitines yields a much larger amount of ATP, be it at a slower rate compared to short-chain acylcarnitines (Figure 7) (Nelson and Cox, 2017a; Talley and Mohiuddin, 2020). Breed differences in lipid metabolism have been reported in the past. An equine training study has reported that there were differences in the lipid utilization between Standardbred and Thoroughbred horses (Assenza et al., 2012). When focusing on the significant high PUFA levels detected in Friesian horses, it is important to mention that these metabolites are necessary for adequate growth, development and general functioning of the adult organism by participating in events such as inhibiting inflammatory processes, decreasing secretion of pro-inflammatory cytokines by monocytes/macrophages and decreasing triglyceride synthesis in the liver (Wiktorowska-Owczarek et al., 2015). Furthermore, in the skeletal muscle, PUFAs can improve mitochondrial function *in vitro*, as well as attenuate age-related declines in mitochondrial protein quality in mice (Casanova et al., 2014; Johnson et al., 2015; Chen et al., 2018). In the past, a significantly increased collagen turnover in Friesian horses has been reported (de Meeûs d’Argenteuil et al., 2021b). The origin of this increased collagen turnover has not been unraveled yet, but it is feasible to assume that this could trigger these increased PUFA levels. More research is needed with that respect.

Finally, the carbohydrate, amino acid and nucleotide super pathways and carnitine metabolism showed significantly higher activity in Warmblood when compared to Standardbred horses. When focusing on the carbohydrate metabolism, TCA cycle activity was more pronounced in Warmblood horses than in Friesians (VL), suggesting the following order: Warmblood > Friesian > Standardbred. The carbohydrate metabolite PEP, an intermediate of glycolysis and gluconeogenesis, had a much lower expression in the VL of Standardbred and Warmblood horses (FD of 0.08 and 0.04) compared to Friesians. PEP is produced from oxaloacetate, which represents the penultimate step of the TCA cycle (Nelson and Cox, 2017b). Interestingly, 3-phosphoglycerate also displayed lower expression in the two other breeds compared to Friesian horses (0.18 and 0.14 FD in Standardbred and Warmblood horses respectively). Galactitol, from the fructose, mannose and galactose metabolic pathway, appeared particularly more abundant in Warmblood horses compared to Friesian horses, especially in the VL. This metabolite is a breakdown product of galactose during an alternative galactose metabolic pathway that is launched upon accumulation of galactose (Jakobs et al., 1995; Zhang et al., 2020). Galactitol can also undergo several changes until fructose-6-phosphate is formed, which is used in the pentose phosphate pathway (PPP) (Kanehisa, 2019; Kanehisa et al., 2021). In the present study fructose-6-phosphate was also more active in Warmblood horses, and the PPP was more active too.

In Friesian horses, the metabolite acetylphosphate, a component of the OXPHOS sub pathway, shows higher activity in the VL compared to Standardbred horses. This suggests that the electron transport chain is more actively involved in aerobic ATP production. (Nelson and Cox, 2017c). Both Standardbred and Warmblood horses displayed in the VL, and in the case of Warmblood horses also in the PM, a greater expression of 2-methylcitrate/homocitrate than Friesian horses. 2-Methylcitrate and homocitrate are intermediates in various metabolic pathways, including the citric acid cycle. Furthermore, 2-methylcitrate can be formed during the metabolism of propionate, a short-chain fatty acid that is produced by the equine microbiome (Kanehisa, 2019; Kanehisa et al., 2021). Aconitate expression is much higher in Warmblood VL muscle when compared to Standardbred horses (3.58 FD) (Table 5). This also stands for advanced glycation end-products ((1.72 FD) for Warmblood > Standardbred) and aminosugar metabolism (1.43 FD Warmblood > Standardbred) (Table 5). Therefore, it seems like metabolites from carbohydrate metabolism are most active in Warmblood horses when compared to Friesian and Standardbred horses. These breed differences in carbohydrate metabolism are more pronounced in the VL when compared to the PM, as expected.

With respect to the amino acid metabolism, Warmblood horses showed significantly higher activity in the VL when compared to Standardbred horses for almost all studied metabolites. However, Friesian horses showed most activity, especially with respect to the BCAA and AAA metabolism. It has been demonstrated that BCAAs bypass the liver and are conveyed to the muscles and that leucine oxidation increases with increasing exercise intensities in humans (McKenzie et al., 2000; Ananieva et al., 2017). The BCAA metabolite alpha-hydroxyisocaproate is also highly active in Friesian horses in both the PM and VL compared to Standardbred and Warmblood horses, and in the VL of Warmblood horses compared to Standardbreds (Supplementary Table S4). This metabolite is known to inhibit protein synthesis and improves muscle recovery (Lang et al., 2013; Sumi et al., 2021). A similar content of metabolites from the BCAA, AAA and other amino acid sub pathways was observed between the PM and VL of Friesian horses. BCAAs may have a regulatory role in the promotion of protein synthesis, as shown in murine and human studies (Kimball and Jefferson, 2006; Brestenský et al., 2015; Mantuano et al., 2020). An example of BCAA that may have an effect on protein turnover in muscle fibers and muscle build-up is pipecolate, which in the present study appeared much higher in Friesian horses in both muscles compared to Warmblood horses (Sato et al., 2016). Pipecolate, is a product of lysine metabolism and its blood levels were found to be lower in Arabian and half-breed Arabian horses after endurance racing (Halama et al., 2021). The amino acid N-(delta)acetylornithine also displays a much higher expression in both muscles of Friesian horses compared to Warmblood horses. This metabolite appears to be synthesized exclusively by the gut microbiota in humans and mice (Liu et al., 2019; Zarei et al., 2021). This suggests that there may be a different baseline diversity of gut microbiota in Friesian horses compared to Warmblood horses, since the food given to them was the same. Taurine and its metabolites such as N-acetyltaurine and taurocyamine are more active in the VL and PM of Warmblood horses compared to Friesian horses. Interestingly, in Arabian and semi-Arabian horses, taurine synthesis increases in response to endurance exercise (Halama et al., 2021). Taurine and its precursors have previously been reported to have antioxidative effects and to support mitochondrial function, which fits the oxidative profile seen in this study in Warmblood horses (Aruoma et al., 1988; Jong et al., 2012). Another AAA that stood out in both muscles of Warmblood horses compared to Friesian horses was cis-urocanate, a metabolite of histidine degradation (Brosnan and Brosnan, 2020) involved in 2-oxoglutarate production, a very important molecule in the TCA cycle (Kanehisa, 2019; Kanehisa et al., 2021). The higher expression of TCA-related metabolites underscores the importance of the TCA cycle in Warmblood horses. It is conceivable that many of the breed-specific highly active amino acid metabolites can act to shape breed-specific energy cycles in this area.

## Conclusion

This study is the first that combines histomorphological parameters with untargeted metabolomics in posture *versus* locomotion muscles in three different archetypical breeds with respect to exercise-type purpose. Though no associations could be found between the involved histomorphological parameters (muscle fiber typing, fCSA and mfCSA) and the untargeted metabolomics profiles, the overarching approach allowed to unveil important breed differences. Moreover, study results show that the simplistic paradigm that small fibers typically rely on slow aerobic metabolism, and large fibers on fast glycolytic based on swift glycogen processing, needs to be carefully interpreted. This interrelation between fiber size and how we think of classical metabolic profiles is probably much more complex than anticipated at this point. This will become more and more clear when performing follow-up training studies in which histomorphological parameters are being combined with untargeted metabolomics. The ST was the least discriminative across breeds and thus seems an interesting muscle to involve in studies where breed differences need to be avoided. Standardbred horses had a significantly higher proportion of type IIA fibers in the PM and VL muscles, while Friesian horses showed a significantly larger representation of type IIX fibers in their PM. No breed differences in fiber specific CSA could be detected, meaning that this feature stands across breeds within the same species. Standardbred horses showed the largest mean CSA of all involved breeds and are predominantly composed of type IIA fibers. The present study clearly shows that metabolic breed differences are more pronounced in locomotion muscles when compared to posture muscles. Linking muscular histological parameters to metabolic output enhances our understanding of muscle function, adaptation, and performance. This knowledge has practical implications in sports science, rehabilitation, and the development of targeted interventions to optimize muscle health and performance. Although in the current study, no associations were found, it should be kept in mind that no grouping of metabolites in main energy cycle groups was performed. This was done on purpose, because for many metabolites this “delineation” per energy cycle is still unclear. Nevertheless, the lipid, amino acid, nucleotide and carbohydrate metabolism show important breed differences in decreasing order. The lipid and nucleotide super pathways showed significantly increased expression in Friesian horses when compared to Standardbreds and Warmblood horses, especially short and medium-chain acylcarnitines. Long and very-long chain acylcarnitines however, were more abundant in Standardbred and Warmblood horses. Also, PUFAs showed significantly higher expression in Friesian horses when compared to other breeds. Friesian horses seem less apt to realize an optimal long and very-long chain fatty acid metabolism, probably correcting for that by importantly involving nucleotide, BCAA and AAA and carbohydrate metabolism. The corner stone of the Warmblood metabolism lays in their carbohydrate, lipid and amino acid metabolism, while Standardbred horses have very high activity in their xenobiotic, lipid and amino acid pathways. The carbohydrate, amino acid and nucleotide super pathways as well as carnitine metabolism showed significantly higher activity in Warmblood when compared to Standardbred horses.

The results of this study indicate that each breed has a distinct metabolic blueprint, conceptualized by centuries of genetic selection towards top performance in certain sports disciplines. Fully mapping the metabolic profile together with the fiber type composition and CSA can support future studies aimed at selecting optimal training regimens and dietary requirements of these breeds to reach their full potential.

## Data Availability

The original contributions presented in the study are included in the article/Supplementary Materials, further inquiries can be directed to the corresponding author.

## Supplementary material

SUPPLEMENTARY FIGURE S1 Bar graph of the average area counts of metabolites for each of the sub pathways found in the *m. vastus lateralis* (VL) of Friesian, Standardbred and Warmblood horses, respectively. Raw area counts were rescaled to set the median equal to 1.

SUPPLEMENTARY FIGURE S2 Bar graph of the average area counts of metabolites for each of the sub pathways found in the *m. pectoralis* (PM) of Friesian and Warmblood horses. Raw area counts were rescaled to set the median equal to 1.

SUPPLEMENTARY TABLE S1 Spearman correlation results between fiber type composition (fiber_type_measurements) and the metabolites found in the *m. pectoralis* (PM), *m. vastus lateralis* (VL) and *m. semitendinosus* (ST) of Friesian (fries) and Standardbred (trotter) horses; spearman_rho represents the significance and spearman_p is the corrected significance to account for the number of comparisons.

SUPPLEMENTARY TABLE S2 Spearman correlation results between fiber cross-sectional area (fiber_type_CSA_measurements) and the metabolites found in the *m. pectoralis* (PM), *m. vastus lateralis* (VL) and *m. semitendinosus* (ST) of Friesian (fries) and Standardbred (trotter) horses; spearman_rho represents the significance and spearman_p is the corrected significance to account for the number of comparisons.

SUPPLEMENTARY TABLE S3 Spearman correlation results between mean fiber cross-sectional area (mean_CSA_measurements) and the metabolites found in the *m. pectoralis* (PM), *m. vastus lateralis* (VL) and *m. semitendinosus* (ST) of Friesian (fries) and Standardbred (trotter) horses; spearman_rho represents the significance and spearman_p is the corrected significance to account for the number of comparisons.

## References

[B1] Adams S. H. Hoppel C. L. Lok K. H. Zhao L. Wong S. W. Minkler P. E. (2009). Plasma acylcarnitine profiles suggest incomplete long-chain fatty acid beta-oxidation and altered tricarboxylic acid cycle activity in type 2 diabetic African-American women. J. Nutr. 139, 1073–1081. 10.3945/jn.108.103754 19369366 PMC2714383

[B2] Adeva-Andany M. M. Calvo-Castro I. Fernández-Fernández C. Donapetry-García C. Pedre-Piñeiro A. M. (2017). Significance of L-carnitine for human health. IUBMB Life 69, 578–594. 10.1002/iub.1646 28653367

[B3] Ahmad Yusof H. Che Muhamed A. M. (2021). Angiotensin-converting enzyme (ACE) insertion/deletion gene polymorphism across ethnicity: a narrative review of performance gene. Sport Sci. Health 17, 57–77. 10.1007/s11332-020-00712-9

[B4] Ananieva E. A. Van Horn C. G. Jones M. R. Hutson S. M. (2017). Liver BCATm transgenic mouse model reveals the important role of the liver in maintaining BCAA homeostasis. J. Nutr. Biochem. 40, 132–140. 10.1016/j.jnutbio.2016.10.014 27886623 PMC5235979

[B5] Aruoma O. I. Halliwell B. Hoey B. M. Butler J. (1988). The antioxidant action of taurine, hypotaurine and their metabolic precursors. Biochem. J. 256, 251–255. 10.1042/bj2560251 2851980 PMC1135395

[B6] Assenza A. Tosto F. Piccione G. Fazio F. Nery J. Valle E. (2012). Lipid utilization pathways induced by early training in Standardbred trotters and Thoroughbreds. J. Equine Vet. Sci. 32, 704–710. 10.1016/j.jevs.2012.02.015

[B7] Benali T. Bakrim S. Ghchime R. Benkhaira N. El Omari N. Balahbib A. (2022). Pharmacological insights into the multifaceted biological properties of quinic acid. Biotechnol. Genet. Eng. Rev. 2022, 1–30. 10.1080/02648725.2022.2122303 36123811

[B8] Bene J. Komlósi K. Gasztonyi B. Juhász M. Tulassay Z. Melegh B. (2005). Plasma carnitine ester profile in adult celiac disease patients maintained on long-term gluten free diet. World J. Gastroenterol. 11, 6671–6675. 10.3748/wjg.v11.i42.6671 16425363 PMC4355763

[B9] Betz M. W. Aussieker T. Kruger C. Q. Gorissen S. H. M. van Loon L. J. C. Snijders T. (2021). Muscle fiber capillarization is associated with various indices of skeletal muscle mass in healthy, older men. Exp. Gerontol. 143, 111161. 10.1016/j.exger.2020.111161 33227401

[B10] Bloemberg D. Quadrilatero J. (2012). Rapid determination of myosin heavy chain expression in rat, mouse, and human skeletal muscle using multicolor immunofluorescence analysis. PLoS One 7, e35273. 10.1371/journal.pone.0035273 22530000 PMC3329435

[B11] Boemer F. Detilleux J. Cello C. Amory H. Marcillaud-Pitel C. Richard E. (2017). Acylcarnitines profile best predicts survival in horses with atypical myopathy. PLoS One 12, e0182761. 10.1371/journal.pone.0182761 28846683 PMC5573150

[B12] Brestenský M. Nitrayová S. Patráš P. Heger J. Nitray J. (2015). Branched chain amino acids and their importance in nutrition. J. Microbiol. Biotechnol. food Sci. 5, 197–202. 10.15414/jmbfs.2015.5.2.197-202

[B13] Brosnan M. E. Brosnan J. T. (2020). Histidine metabolism and function. J. Nutr. 150, 2570S–2575S. 10.1093/jn/nxaa079 33000155 PMC7527268

[B14] Buechler K. F. Lowenstein J. M. (1990). The involvement of carnitine intermediates in peroxisomal fatty acid oxidation: a study with 2-bromofatty acids. Arch. Biochem. Biophys. 281, 233–238. 10.1016/0003-9861(90)90437-4 2393299

[B15] Cao G. Song Z. Hong Y. Yang Z. Song Y. Chen Z. (2020). Large-scale targeted metabolomics method for metabolite profiling of human samples. Anal. Chim. Acta 1125, 144–151. 10.1016/j.aca.2020.05.053 32674760

[B16] Casanova E. Baselga-Escudero L. Ribas-Latre A. Arola-Arnal A. Bladé C. Arola L. (2014). Epigallocatechin gallate counteracts oxidative stress in docosahexaenoxic acid-treated myocytes. Biochim. Biophys. Acta - Bioenerg. 1837, 783–791. 10.1016/j.bbabio.2014.01.014 24486445

[B17] Chen P. B. Yang J. S. Park Y. (2018). Adaptations of skeletal muscle mitochondria to obesity, exercise, and polyunsaturated fatty acids. Lipids 53, 271–278. 10.1002/lipd.12037 29663395

[B18] Choi J. Y. Lee J. W. Jang H. Kim J. G. Lee M. K. Hong J. T. (2021). Quinic acid esters from Erycibe obtusifolia with antioxidant and tyrosinase inhibitory activities. Nat. Prod. Res. 35, 3026–3032. 10.1080/14786419.2019.1684285 31680567

[B19] Cosgrove E. J. Sadeghi R. Schlamp F. Holl H. M. Moradi-Shahrbabak M. Miraei-Ashtiani S. R. (2020). Genome diversity and the origin of the arabian horse. Sci. Rep. 10, 9702–9713. 10.1038/s41598-020-66232-1 32546689 PMC7298027

[B20] Dambrova M. Makrecka-Kuka M. Kuka J. Vilskersts R. Nordberg D. Attwood M. M. (2022). Acylcarnitines: nomenclature, biomarkers, therapeutic potential, drug targets, and clinical trials. Pharmacol. Rev. 74, 506–551. 10.1124/pharmrev.121.000408 35710135

[B21] de Meeûs d’Argenteuil C. Boshuizen B. Oosterlinck M. van de Winkel D. De Spiegelaere W. de Bruijn C. M. (2021a). Flexibility of equine bioenergetics and muscle plasticity in response to different types of training: an integrative approach, questioning existing paradigms. PLoS One 16, e0249922. 10.1371/journal.pone.0249922 33848308 PMC8043414

[B22] Dechick A. Hetz R. Lee J. Speelman D. L. (2020). Increased skeletal muscle fiber cross-sectional area, muscle phenotype shift, and altered insulin signaling in rat hindlimb muscles in a prenatally androgenized rat model for polycystic ovary syndrome. Int. J. Mol. Sci. 21, 7918–7924. 10.3390/ijms21217918 33113794 PMC7662395

[B23] Dehaven C. D. Evans A. M. Dai H. Lawton K. A. (2010). Organization of GC/MS and LC/MS metabolomics data into chemical libraries. J. Cheminform. 2, 9–12. 10.1186/1758-2946-2-9 20955607 PMC2984397

[B24] de Meeûs d’Argenteuil C. Boshuizen B. Vidal Moreno de Vega C. Leybaert L. de Maré L. Goethals K. (2021b). Comparison of shifts in skeletal muscle plasticity parameters in horses in three different muscles, in answer to 8 weeks of harness training. Front. Vet. Sci. 8, 718866. 10.3389/fvets.2021.718866 34733900 PMC8558477

[B25] Engelking L. R. (2015). “Fatty acid oxidation,” in Textbook of veterinary physiological chemistry. Editor Engelking E (Boston: Academic Press), 351–357. 10.1016/B978-0-12-391909-0.50055-4

[B26] Essén B. Lindholm A. Thornton J. (1980). Histochemical properties of muscle fibres types and enzyme activities in skeletal muscles of Standardbred trotters of different ages. Equine Vet. J. 12, 175–180. 10.1111/j.2042-3306.1980.tb03420.x 6449365

[B27] Evans A. M. DeHaven C. D. Barrett T. Mitchell M. Milgram E. (2009). Integrated, nontargeted ultrahigh performance liquid chromatography/electrospray ionization tandem mass spectrometry platform for the identification and relative quantification of the small-molecule complement of biological systems. Anal. Chem. 81, 6656–6667. 10.1021/ac901536h 19624122

[B28] Ferdinandusse S. Denis S. Van Roermund C. W. T. Wanders R. J. A. Dacremont G. (2004). Identification of the peroxisomal beta-oxidation enzymes involved in the degradation of long-chain dicarboxylic acids. J. Lipid Res. 45, 1104–1111. 10.1194/jlr.M300512-JLR200 15060085

[B29] Firsham A. M. Valberg S. J. Baird J. D. Hunt L. DiMauro S. (2008). Insulin sensitivity in Belgian horses with polysaccharide storage myopathy. Am. J. Vet. Res. 69, 818–823. 10.2460/ajvr.69.6.818 18518664

[B30] Fulghum K. Collins H. E. Jones S. P. Hill B. G. (2022). Influence of biological sex and exercise on murine cardiac metabolism. J. Sport Heal. Sci. 11, 479–494. 10.1016/j.jshs.2022.06.001 PMC9338340 35688382

[B31] Gejl K. D. Hvid L. G. Andersson E. P. Jensen R. Holmberg H. C. Ørtenblad N. (2021). Contractile properties of MHC I and II fibers from highly trained arm and leg muscles of cross-country skiers. Front. Physiol. 12, 682943–683010. 10.3389/fphys.2021.682943 34220547 PMC8242206

[B32] Giacomello E. Crea E. Torelli L. Bergamo A. Reggiani C. Sava G. (2020). Age dependent modification of the metabolic profile of the tibialis anterior muscle fibers in C57BL/6J mice. Int. J. Mol. Sci. 21, 3923. 10.3390/ijms21113923 32486238 PMC7312486

[B33] Grotmol S. Totland G. K. Kryvi H. Breistøl A. Essén-Gustavsson B. Lindholm A. (2002). Spatial distribution of fiber types within skeletal muscle fascicles from standardbred horses. Anat. Rec. 268, 131–136. 10.1002/ar.10140 12221719

[B34] Gunn H. M. (1987). “Muscle, bone and fat proportions and muscle distribution of thoroughbreds and other horses,” in Proc. 2nd ICEEP Equine Exercise Physiology. Editors Gillespie J. R. Robinson N. E. (ICEEP, 1988), 253–264.

[B35] Halama A. Horsch M. Kastenmüller G. Möller G. Kumar P. Prehn C. (2016). Metabolic switch during adipogenesis: from branched chain amino acid catabolism to lipid synthesis. Arch. Biochem. Biophys. 589, 93–107. 10.1016/j.abb.2015.09.013 26408941

[B36] Halama A. Oliveira J. M. Filho S. A. Qasim M. Achkar I. W. Johnson S. (2021). Metabolic predictors of equine performance in endurance racing. Metabolites 11, 82–18. 10.3390/metabo11020082 33572513 PMC7912089

[B37] Hall E. C. R. Semenova E. A. Bondareva E. A. Borisov O. V. Andryushchenko O. N. Andryushchenko L. B. (2021). Association of muscle fiber composition with health and exercise-related traits in athletes and untrained subjects. Biol. Sport 38, 659–666. 10.5114/biolsport.2021.102923 34937976 PMC8670815

[B38] Hao J. W. Wang J. Guo H. Zhao Y. Y. Sun H. H. Li Y. F. (2020). CD36 facilitates fatty acid uptake by dynamic palmitoylation-regulated endocytosis. Nat. Commun. 11, 4765–4816. 10.1038/s41467-020-18565-8 32958780 PMC7505845

[B39] Harpalani V. (2017). “The athletic dominance of African Americans—is there a genetic basis?,” in In contemporary themes: african Americans in sport (Oxfordshire: Routledge), 103–120. 10.1007/s12111-996-1003-6

[B40] Hoppel C. (2003). The role of carnitine in normal and altered fatty acid metabolism. Am. J. Kidney Dis. 41, S4–S12. 10.1016/S0272-6386(03)00112-4 12751049

[B41] Islas A. Lopez-Rivero J. L. Quezada M. Mora G. Merino V. Briones M. (1997). Características histoquímicas y bioquímicas de las fibras del músculo Gluteus medius en equinos de tiro descendientes del plan de fomento equino. Arch. Med. Vet. 29, 35–43. 10.4067/s0301-732x1997000100004

[B42] Jacob M. Malkawi A. Albast N. Al Bougha S. Lopata A. Dasouki M. (2018). A targeted metabolomics approach for clinical diagnosis of inborn errors of metabolism. Anal. Chim. Acta 1025, 141–153. 10.1016/j.aca.2018.03.058 29801603

[B43] Jakobs C. Schweitzer S. Dorland B. (1995). Galactitol in galactosemia. Eur. J. Pediatr. 154, S50–S52. 10.1007/BF02143804 7671965

[B44] Jeon Y. Choi J. Kim H. J. Lee H. Lim J. Y. Choi S. J. (2019). Sex- and fiber type-related contractile properties in human single muscle fiber. J. Exerc. Rehabil. 15, 537–545. 10.12965/jer.1938336.168 31523674 PMC6732543

[B45] Johnson M. L. Lalia A. Z. Dasari S. Pallauf M. Fitch M. Hellerstein M. K. (2015). Eicosapentaenoic acid but not docosahexaenoic acid restores skeletal muscle mitochondrial oxidative capacity in old mice. Aging Cell 14, 734–743. 10.1111/acel.12352 26010060 PMC4568961

[B46] Jong C. J. Azuma J. Schaffer S. (2012). Mechanism underlying the antioxidant activity of taurine: prevention of mitochondrial oxidant production. Amino Acids 42, 2223–2232. 10.1007/s00726-011-0962-7 21691752

[B47] Kanehisa M. (2019). Toward understanding the origin and evolution of cellular organisms. Protein Sci. 28, 1947–1951. 10.1002/pro.3715 31441146 PMC6798127

[B48] Kanehisa M. Furumichi M. Sato Y. Ishiguro-Watanabe M. Tanabe M. (2021). KEGG: integrating viruses and cellular organisms. Nucleic Acids Res. 49, D545–D551. 10.1093/nar/gkaa970 33125081 PMC7779016

[B49] Karlstrom K. Essen-Gustavsson B. Lindholm A. (1994). Fibre type distribution, capillarization and enzymatic profile of locomotor and nonlocomotor muscles of horses and steers. Acta Anat. (Basel). 151, 97–106. 10.1159/000147649 7701935

[B50] Kauter A. Epping L. Semmler T. Antao E.-M. Kannapin D. Stoeckle S. D. (2019). The gut microbiome of horses: current research on equine enteral microbiota and future perspectives. Anim. Microbiome 1, 14–15. 10.1186/s42523-019-0013-3 33499951 PMC7807895

[B51] Kimball S. R. Jefferson L. S. (2006). Signaling pathways and molecular mechanisms through which branched-chain amino acids mediate translational control of protein synthesis. J. Nutr. 136, 227S–231S. 10.1093/jn/136.1.227s 16365087

[B52] Klein B. G. (2020). “The physiology of muscle,” in Cunninham’s textbook of veterinary physiology (St.Louis: Elsevier), 73–81.

[B53] Klein D. J. McKeever K. H. Mirek E. T. Anthony T. G. (2020). Metabolomic response of equine skeletal muscle to acute fatiguing exercise and training. Front. Physiol. 11, 110–115. 10.3389/fphys.2020.00110 32132934 PMC7040365

[B54] Kohn T. A. Burroughs R. Hartman M. J. Noakes T. D. (2011). Fiber type and metabolic characteristics of lion (*Panthera leo*), caracal (Caracal caracal) and human skeletal muscle. Comp. Biochem. Physiol. - A Mol. Integr. Physiol. 159, 125–133. 10.1016/j.cbpa.2011.02.006 21320626

[B55] Lang C. H. Pruznak A. Navaratnarajah M. Rankine K. A. Deiter G. Magne H. (2013). Chronic α-hydroxyisocaproic acid treatment improves muscle recovery after immobilization-induced atrophy. Am. J. Physiol. - Endocrinol. Metab. 305, 416–428. 10.1152/ajpendo.00618.2012 23757407

[B56] Larson L. Lioy J. Johnson J. Medler S. (2019). Transitional hybrid skeletal muscle fibers in rat soleus development. J. Histochem. Cytochem. 67, 891–900. 10.1369/0022155419876421 31510854 PMC6882066

[B57] Latham C. M. White S. H. (2017). 107 Validation of primary antibodies for multiple immunofluorescent labeling of horse skeletal muscle fiber type. J. Anim. Sci. 95, 53. 10.2527/asasann.2017.107 28177358

[B58] Leckey J. J. Hoffman N. J. Parr E. B. Devlin B. L. Trewin A. J. Stepto N. K. (2018). High dietary fat intake increases fat oxidation and reduces skeletal muscle mitochondrial respiration in trained humans. FASEB J. 32, 2979–2991. 10.1096/fj.201700993R 29401600

[B59] Li H. F. Shu J. T. Shan Y. J. Chen W. F. Song C. Xu W. J. (2016). Myofiber development during embryonic to neonatal development in duck breeds differing in muscle growth rates. J. Integr. Agric. 15, 403–413. 10.1016/S2095-3119(14)60949-7

[B60] Lievens E. Bellinger P. Van Vossel K. Vancompernolle J. Bex T. Minahan C. (2021a). Muscle typology of world-class cyclists across various disciplines and events. Med. Sci. Sports Exerc. 53, 816–824. 10.1249/MSS.0000000000002518 33105386

[B61] Lievens E. Klass M. Bex T. Derave W. (2020). Muscle fiber typology substantially influences time to recover from highintensity exercise. J. Appl. Physiol. 128, 648–659. 10.1152/japplphysiol.00636.2019 31999527

[B62] Lievens E. Van Vossel K. Van De Casteele F. Krssak M. Murdoch J. B. Befroy D. E. (2021b). CORP: quantification of human skeletal muscle carnosine concentration by proton magnetic resonance spectroscopy. J. Appl. Physiol. 131, 250–264. 10.1152/japplphysiol.00056.2021 33982593

[B63] Lievens E. Van Vossel K. Van de Casteele F. Wezenbeek E. Deprez D. Matthys S. (2022). Muscle fibre typology as a novel risk factor for hamstring strain injuries in professional football (soccer): a prospective cohort study. Sport. Med. 52, 177–185. 10.1007/s40279-021-01538-2 34515974

[B64] Lindner A. Dag Erginsoy S. Kissenbeck S. Mosen H. Hetzel U. Drommer W. (2013). Effect of different blood-guided conditioning programmes on skeletal muscle ultrastructure and histochemistry of sport horses. J. Anim. Physiol. Anim. Nutr. Berl. 97, 374–386. 10.1111/j.1439-0396.2012.01283.x 22404305

[B65] Liu Y. Tian X. He B. Hoang T. K. Taylor C. M. Blanchard E. (2019). Lactobacillus reuteri DSM 17938 feeding of healthy newborn mice regulates immune responses while modulating gut microbiota and boosting beneficial metabolites. Am. J. Physiol. - Gastrointest. Liver Physiol. 317, G824–G838. 10.1152/ajpgi.00107.2019 31482733 PMC6962498

[B66] López-Rivero J. L. Agüera E. Monterde J. G. Rodríguez-Barbudo M. V. Miró F. (1989). Comparative study of muscle fiber type composition in the middle gluteal muscle of andalusian, thoroughbred and arabian horses. J. Equine Vet. Sci. 9, 337–340. 10.1016/S0737-0806(89)80072-3

[B67] López-Rivero J. L. Morales-Lopez J. L. Galisteo A. M. Aguera E. (1991). Muscle fibre type composition in untrained and endurance‐trained Andalusian and Arab horses. Equine Vet. J. 23, 91–93. 10.1111/j.2042-3306.1991.tb02727.x 2044515

[B68] Mach N. Moroldo M. Rau A. Lecardonnel J. Le Moyec L. Robert C. (2021). Understanding the holobiont: crosstalk between gut microbiota and mitochondria during long exercise in horse. Front. Mol. Biosci. 8, 656204. 10.3389/fmolb.2021.656204 33898524 PMC8063112

[B69] Mantuano P. Bianchini G. Cappellari O. Boccanegra B. Conte E. Sanarica F. (2020). Ergogenic effect of bcaas and l-alanine supplementation: proof-of-concept study in a murine model of physiological exercise. Nutrients 12, 2295–2324. 10.3390/nu12082295 32751732 PMC7468919

[B70] Marín Navas C. Delgado Bermejo J. V. McLean A. K. León Jurado J. M. Rodriguez de la Borbolla y Ruiberriz de Torres A. Navas González F. J. (2021). Discriminant canonical analysis of the contribution of Spanish and arabian purebred horses to the genetic diversity and population structure of hispano-arabian horses. Animals 11, 269–327. 10.3390/ani11020269 33494478 PMC7912545

[B71] McKenzie S. Phillips S. M. Carter S. L. Lowther S. Gibala M. J. Tarnopolsky M. A. (2000). Endurance exercise training attenuates leucine oxidation and BCOAD activation during exercise in humans. Am. J. Physiol. - Endocrinol. Metab. 278, E580–E587. 10.1152/ajpendo.2000.278.4.e580 10751189

[B72] Meng S. Zhang Y. Lv S. Zhang Z. Liu X. Jiang L. (2023). Comparison of muscle metabolomics between two Chinese horse breeds. Front. Vet. Sci. 10, 1162953. 10.3389/fvets.2023.1162953 37215482 PMC10196265

[B73] Metaxas T. Mandroukas A. Michailidis Y. Koutlianos N. Christoulas K. Ekblom B. (2019). Correlation of fiber-type composition and sprint performance in youth soccer players. J. Strength Cond. Res. 33, 2629–2634. 10.1519/JSC.0000000000003320 31403577

[B74] Miles F. L. Orlich M. J. Mashchak A. Chandler P. D. Lampe J. W. Duerksen-Hughes P. (2022). The biology of veganism: plasma metabolomics analysis reveals distinct profiles of vegans and non-vegetarians in the adventist health study-2 cohort. Nutrients 14, 709. 10.3390/nu14030709 35277064 PMC8839915

[B75] Miller M. S. Bedrin N. G. Ades P. A. Palmer B. M. Toth M. J. (2015). Molecular determinants of force production in human skeletal muscle fibers: effects of myosin isoform expression and cross-sectional area. Am. J. Physiol. - Cell Physiol. 308, C473–C484. 10.1152/ajpcell.00158.2014 25567808 PMC4360030

[B76] Moro T. Brightwell C. R. Phalen D. E. McKenna C. F. Lane S. J. Porter C. (2019). Low skeletal muscle capillarization limits muscle adaptation to resistance exercise training in older adults. Exp. Gerontol. 127, 110723. 10.1016/j.exger.2019.110723 31518665 PMC6904952

[B77] Nam S. Y. Han N. R. Rah S. Y. Seo Y. Kim H. M. Jeong H. J. (2018). Anti-inflammatory effects of Artemisia scoparia and its active constituent, 3,5-dicaffeoyl-epi-quinic acid against activated mast cells. Immunopharmacol. Immunotoxicol. 40, 52–58. 10.1080/08923973.2017.1405438 29172841

[B78] Nelson D. L. Cox M. M. (2017a). “Fatty acid catabolism,” in Lehninger principles of biochemistry. Editors Schultz L Moran S Moloney S (Basingstoke: MacMillan Higher Education), 647–672.

[B79] Nelson D. L. Cox M. M. (2017b). “Glycolysis, gluconeogenesis and the pentose phosphate pathway,” in Lehninger principles of biochemistry. Editors Schultz L Moran S Moloney S (Basingstoke: MacMillan Higher Education), 533–574.

[B80] Nelson D. L. Cox M. M. (2017c). “Oxidative phosphorylation,” in Lehninger principles of biochemistry. Editors Schultz L Moran S Moloney S (Basingstoke: MacMillan Higher Education), 707–750.

[B81] Nyerges-Bohák Z. Nagy K. Rózsa L. Póti P. Kovács L. (2021). Heart rate variability before and after 14 weeks of training in Thoroughbred horses and Standardbred trotters with different training experience. PLoS One 16, 02599333. 10.1371/journal.pone.0259933 PMC8659354 34882704

[B82] Payne R. C. Hutchinson J. R. Robilliard J. J. Smith N. C. Wilson A. M. (2005a). Functional specialisation of pelvic limb anatomy in horses (*Equus caballus*). J. Anat. 206, 557–574. 10.1111/j.1469-7580.2005.00420.x 15960766 PMC1571521

[B83] Payne R. C. Veenman P. Wilson A. M. (2005b). Erratum: the role of the extrinsic thoracic limb muscles in equine locomotion (Journal of Anatomy (2004)). J. Anat. 206, 193–204. 10.1111/j.1469-7580.2005.00353.x 15730484 PMC1571467

[B84] Przewłócka K. Folwarski M. Kaźmierczak-Siedlecka K. Skonieczna-żydecka K. Kaczor J. J. (2020). Gut-muscle AxisExists and may affect skeletal muscle adaptation to training. Nutrients 12, 1451–1519. 10.3390/nu12051451 32443396 PMC7285193

[B85] Rietbroek N. J. Dingboom E. G. Joosten B. J. L. J. Eizema K. Everts M. E. (2007). Effect of show jumping training on the development of locomotory muscle in young horses. Am. J. Vet. Res. 68, 1232–1238. 10.2460/ajvr.68.11.1232 17975979

[B86] Ringmark S. Jansson A. Lindholm A. Hedenström U. Roepstorff L. (2016). A 2.5 year study on health and locomotion symmetry in young Standardbred horses subjected to two levels of high intensity training distance. Vet. J. 207, 99–104. 10.1016/j.tvjl.2015.10.052 26654845

[B87] Rivero J.-L. L. Diz A. M. (1992). Skeletal muscle histochemistry of Andalusian horses: a comparative study with other breeds. Arch. Zootec. 41, 505–512.

[B88] Rivero J. L. L. Hill E. W. (2016). Skeletal muscle adaptations and muscle genomics of performance horses. Vet. J. 209, 5–13. 10.1016/J.TVJL.2015.11.019 26831154

[B89] Rivero J. L. L. Ruz M. C. Serrano A. L. Diz A. M. (1995). Effects of a 3 month endurance training programme on skeletal muscle histochemistry in Andalusian, Arabian and Anglo‐Arabian horses. Equine Vet. J. 27, 51–59. 10.1111/j.2042-3306.1995.tb03033.x 7774548

[B90] Rivero J. L. L. Serrano A. L. Henckel P. Aguera E. (1993). Muscle fiber type composition and fiber size in successfully and unsuccessfully endurance-raced horses. J. Appl. Physiol. 75, 1758–1766. 10.1152/jappl.1993.75.4.1758 8282629

[B91] Robertson C. E. McClelland G. B. (2019). Developmental delay in shivering limits thermogenic capacity in juvenile high-altitude deer mice (*Peromyscus maniculatus*). J. Exp. Biol. 222, jeb210963. 10.1242/jeb.210963 31562187

[B92] Rodríguez-Sánchez I. P. Treviño-Alvarado V. M. del Rosario Torres-Sepúlveda M. López-Saldaña L. A. Ponce-García G. López-Uriarte G. A. (2015). Reference values for amino acids and acylcarnitines in peripheral blood in Quarter horses and American Miniature horses. Acta Vet. Scand. 57, 62. 10.1186/s13028-015-0144-9 26416518 PMC4587867

[B93] Saito M. Ginszt M. Semenova E. Massidda M. Huminska-Lisowska K. Michałowska-Sawczyn M. (2022). Genetic profile of sports climbing athletes from three different ethnicities. Biol. Sport 39, 913–919. 10.5114/biolsport.2022.109958 36247943 PMC9536361

[B94] Sato T. Ito Y. Nagasawa T. (2016). Regulatory effects of the L-lysine metabolites, L-2-aminoadipic acid and L-pipecolic acid, on protein turnover in C2C12 myotubes. Biosci. Biotechnol. Biochem. 80, 2168–2175. 10.1080/09168451.2016.1210499 27427787

[B95] Schiaffino S. Reggiani C. (2011). Fiber types in mammalian skeletal muscles. Physiol. Rev. 91, 1447–1531. 10.1152/physrev.00031.2010 22013216

[B96] Serrano A. L. Rivero J. L. L. (2000). Myosin heavy chain profile of equine gluteus medius muscle following prolonged draught-exercise training and detraining. J. Muscle Res. Cell Motil. 21, 235–245. 10.1023/A:1005642632711 10952171

[B97] Snow D. H. Guy P. S. (1980). Muscle fibre type composition of a number of limb muscles in different types of horse. Res. Vet. Sci. 28, 137–144. 10.1016/S0034-5288(18)32735-8 6447905

[B98] Srisawat K. Shepherd S. Lisboa P. Burniston J. (2017). A systematic review and meta-analysis of proteomics literature on the response of human skeletal muscle to obesity/type 2 diabetes mellitus (T2DM) versus exercise training. Proteomes 5, 30. 10.3390/proteomes5040030 29137117 PMC5748565

[B99] Sumi K. Sakuda M. Munakata K. Nakamura K. Ashida K. (2021). α-Hydroxyisocaproic acid decreases protein synthesis but attenuates TNFα/IFNγ co-exposure-induced protein degradation and myotube atrophy via suppression of iNOS and IL-6 in murine C2C12 myotube. Nutrients 13, 2391. 10.3390/nu13072391 34371902 PMC8308709

[B100] Talley J. T. Mohiuddin S. S. (2020). Biochemistry, fatty acid oxidation. StatPearls Publishing, Available at: https://www.ncbi.nlm.nih.gov/books/NBK556002/ (Accessed July 3, 2023). 32310462

[B101] Tan J. McKenzie C. Potamitis M. Thorburn A. N. Mackay C. R. Macia L. (2014). The role of short-chain fatty acids in health and disease. Adv. Immunol. 121, 91–119. 10.1016/B978-0-12-800100-4.00003-9 24388214

[B102] Taylor A. W. Brassard L. (1981). Skeletal muscle fiber distribution and area in trained and stalled standardbred horses. Can. J. Anim. Sci. 61, 601–605. 10.4141/cjas81-072

[B103] Thienen R. V. D’Hulst G. Deldicque L. Hespel P. (2014). Biochemical artifacts in experiments involving repeated biopsies in the same muscle. Physiol. Rep. 2, e00286. 10.14814/phy2.286 24819751 PMC4098731

[B104] Tullson P. C. Terjung R. L. (1991). Adenine nucleotide synthesis in exercising and endurance-trained skeletal muscle. Am. J. Physiol. - Cell Physiol. 261, C342–C347. 10.1152/ajpcell.1991.261.2.c342 1908187

[B105] Tyler C. M. Golland L. C. Evans D. L. Hodgson D. R. Rose R. J. (1998). Skeletal muscle adaptations to prolonged training, overtraining and detraining in horses. Pflugers Arch. Eur. J. Physiol. 436, 391–397. 10.1007/s004240050648 9644221

[B106] Valberg S. (1987). Metabolic response to racing and fiber properties of skeletal muscle in standardbred and thoroughbred horses. J. Equine Vet. Sci. 7, 6–12. 10.1016/S0737-0806(87)80085-0

[B107] Valberg S. J. Iglewski H. Henry M. L. Schultz A. E. McKenzie E. C. (2022). Skeletal muscle fiber type composition and citrate synthase activity in fit and unfit Warmbloods and Quarter horses. J. Equine Vet. Sci. 118, 104123. 10.1016/j.jevs.2022.104123 36096315

[B108] van Boom K. M. Breed D. Hughes A. Blackhurst D. Kohn T. A. (2022). A novel description of the Vastus lateralis morphology of the Temminck’s ground pangolin (Manis temminckii). Anat. Rec. 305, 3463–3471. 10.1002/ar.24924 35357087

[B109] van Boom K. M. Schoeman J. P. Steyl J. C. A. Kohn T. A. (2023). Fiber type and metabolic characteristics of skeletal muscle in 16 breeds of domestic dogs. Anat. Rec. 306 (10), 2572–2586. 10.1002/ar.25207 36932662

[B110] van der Kolk J. H. Thomas S. Mach N. Ramseyer A. Burger D. Gerber V. (2020). Serum acylcarnitine profile in endurance horses with and without metabolic dysfunction. Vet. J. 255, 105419. 10.1016/j.tvjl.2019.105419 31982078

[B111] van Ginneken M. M. E. de Graaf-Roelfsema E. Keizer H. A. VanDam K. G. Wijnberg I. D. van der Kolk J. H. (2006). Immunohistochemical identification and fiber type specific localization of protein kinase C isoforms in equine skeletal muscle. Am. J. Vet. Res. 67, 69–73. 10.2460/ajvr.2004.65.69 14719705

[B112] Van Wessel T. De Haan A. Van Der Laarse W. J. Jaspers R. T. (2010). The muscle fiber type-fiber size paradox: hypertrophy or oxidative metabolism? Eur. J. Appl. Physiol. 110, 665–694. 10.1007/s00421-010-1545-0 20602111 PMC2957584

[B113] Vissing K. Andersen J. L. Schjerling P. (2005). Are exercise‐induced genes induced by exercise? FASEB J. 19, 94–96. 10.1096/fj.04-2084fje 15516373

[B114] Warren B. E. Lou P. H. Lucchinetti E. Zhang L. Clanachan A. S. Affolter A. (2014). Early mitochondrial dysfunction in glycolytic muscle, but not oxidative muscle, of the fructose-fed insulin-resistant rat. Am. J. Physiol. - Endocrinol. Metab. 306, 658–667. 10.1152/ajpendo.00511.2013 PMC3948982 24425766

[B115] Westermann C. M. Dorland B. de Sain-van der Velden M. G. Wijnberg I. D. van Breda E. de Graaf-Roelfsema E. (2008). Plasma acylcarnitine and fatty acid profiles during exercise and training in Standardbreds. Am. J. Vet. Res. 69, 1469–1475. 10.2460/ajvr.69.11.1469 18980429

[B116] Wiktorowska-Owczarek A. Berezińska M. Nowak J. Z. (2015). PUFAs: structures, metabolism and functions. Adv. Clin. Exp. Med. 24, 931–941. 10.17219/acem/31243 26771963

[B117] Wishart D. S. Feunang Y. D. Marcu A. Guo A. C. Liang K. Vázquez-Fresno R. (2018). HMDB 4.0: the human metabolome database for 2018. Nucleic Acids Res. 46, D608–D617. 10.1093/nar/gkx1089 29140435 PMC5753273

[B118] Zarei I. Baxter B. A. Oppel R. C. Borresen E. C. Brown R. J. Ryan E. P. (2021). Plasma and urine metabolite profiles impacted by increased dietary navy bean intake in colorectal cancer survivors: a randomized-controlled trial. Cancer Prev. Res. 14, 497–508. 10.1158/1940-6207.CAPR-20-0270 PMC8026539 33361317

[B119] Zhang W. Chen J. Chen Q. Wu H. Mu W. (2020). Sugar alcohols derived from lactose: lactitol, galactitol, and sorbitol. Appl. Microbiol. Biotechnol. 104, 9487–9495. 10.1007/s00253-020-10929-w 32989517

