# Supplementary figures and images for: Baselining physiological parameters in three muscles across three equine breeds. What can we learn from the horse?
Source: Front Physiol. 2024 Feb 7;15:1291151. doi: 10.3389/fphys.2024.1291151 (PMC10879303; doi:10.3389/fphys.2024.1291151)

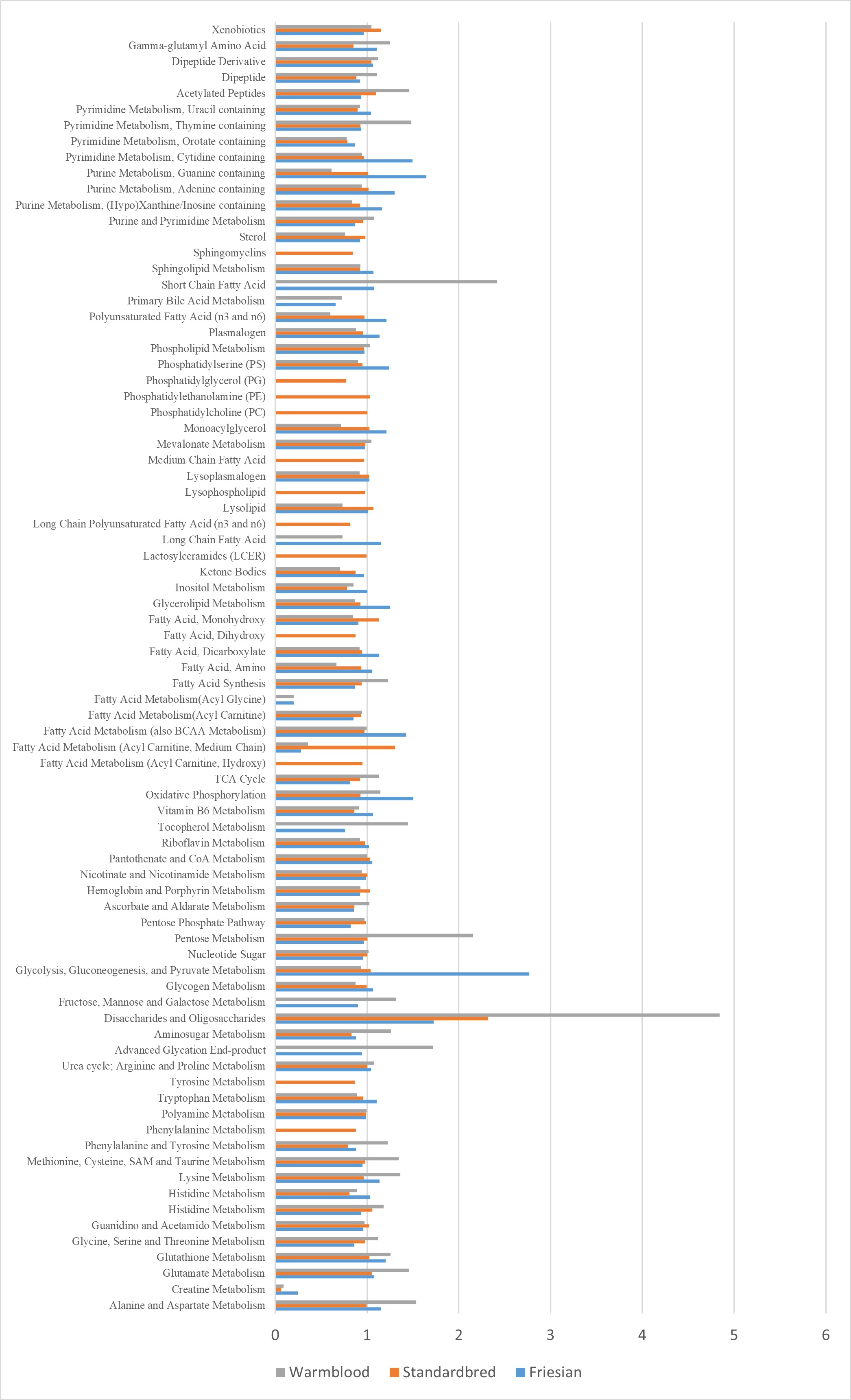

Supplement: Supplementary file 3 [file Image1.JPEG]

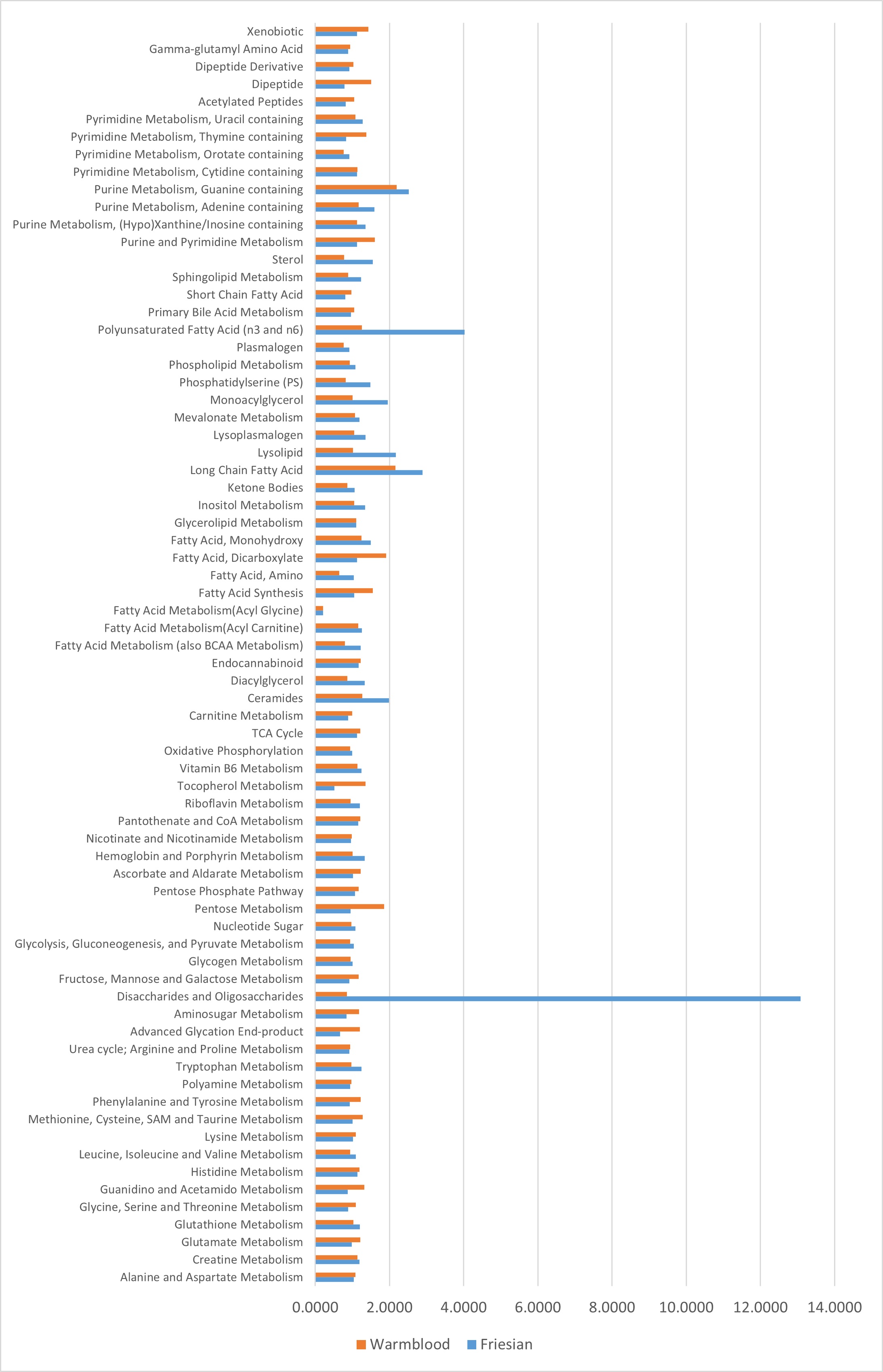

Supplement: Supplementary file 4 [file Image2.JPEG]
