# Supplementary material for: Baselining physiological parameters in three muscles across three equine breeds. What can we learn from the horse?
Source: Front Physiol. 2024 Feb 7;15:1291151. doi: 10.3389/fphys.2024.1291151 (PMC10879303; doi:10.3389/fphys.2024.1291151)
Supplement: Supplementary file 6 [file Table4.docx]

Supplementary Table 4: Summary of metabolites found significantly different in the baseline metabolic profile of the *m. pectoralis* (PM) and the *m. vastus lateralis* (VL) between Warmblood and Friesian horses with their respective fold difference (FD). Legend: dark red: values over 2-FD; light red: values equal to 1 and lower than 2-FD; dark green: values below 0.5-FD; light green: values between 0.5 and 1-FD; blank cells correspond to metabolites that were not found significant or existent across the compared breeds. For both muscles (PM and VL), table cells in red represent metabolites more active in Warmblood horses and cells in green represent metabolites more active in Friesian horses.

| **Superpathway** | **Subpathway** | **Metabolite** | **PM**  **(Fold difference)** | **VL**  **(Fold difference)** |
| --- | --- | --- | --- | --- |
| Lipid | Carnitine Metabolism | deoxycarnitine | 1.11 | 1 |
|  |  | carnitine | 1.14 | 1.18 |
|  | Fatty Acid Metabolism (also BCAA Metabolism) | butyrylcarnitine (C4) | 0.53 | 0.55 |
|  |  | propionylcarnitine (C3) | 0.79 | 0.89 |
|  | Fatty Acid Metabolism(Acyl Carnitine) | acetylcarnitine (C2) | 0.86 | 0.89 |
|  |  | 3-hydroxybutyrylcarnitine | 0.73 | 0.84 |
|  |  | hexanoylcarnitine (C6) | 0.54 | 0.76 |
|  |  | octanoylcarnitine (C8) | 0.59 | 0.8 |
|  |  | decanoylcarnitine (C10) | 0.67 | 0.74 |
|  |  | cis-4-decenoylcarnitine (C10:1) | 0.75 | 0.93 |
|  |  | laurylcarnitine (C12) | 0.86 | 0.75 |
|  |  | myristoylcarnitine (C14) | 0.72 | 0.78 |
|  |  | palmitoylcarnitine (C16) | 0.86 | 0.89 |
|  |  | palmitoleoylcarnitine (C16:1)* | 0.81 | 0.9 |
|  |  | stearoylcarnitine (C18) | 0.85 | 1.17 |
|  |  | linoleoylcarnitine (C18:2)* | 1.18 | 1.36 |
|  |  | linolenoylcarnitine (C18:3)* | 0.98 | 1.19 |
|  |  | oleoylcarnitine (C18:1) | 1.11 | 1.24 |
|  |  | myristoleoylcarnitine (C14:1)* | 0.62 | 0.71 |
|  |  | adipoylcarnitine (C6-DC) | 1.32 | 1.16 |
|  |  | arachidoylcarnitine (C20)* | 1.05 | 1.35 |
|  |  | arachidonoylcarnitine (C20:4) | 1.38 | 2.01 |
|  |  | adrenoylcarnitine (C22:4)* | 1.39 | 2.48 |
|  |  | dihomo-linolenoylcarnitine (20:3n3 or 6)* | 1.1 | 2.09 |
|  |  | dihomo-linoleoylcarnitine (C20:2)* | 1.05 | 1.57 |
|  |  | eicosenoylcarnitine (C20:1)* | 1.13 | 1.7 |
|  |  | erucoylcarnitine (C22:1)* | 1.06 | 1.47 |
|  |  | docosatrienoylcarnitine (C22:3)* | 1.21 | 1.81 |
|  |  | docosapentaenoylcarnitine (C22:5n3)* | 1.51 | 2.22 |
|  |  | docosahexaenoylcarnitine (C22:6)* | 1.13 | 1.56 |
|  |  | margaroylcarnitine* | 1.01 | 1.19 |
|  |  | pentadecanoylcarnitine (C15)* | 0.86 | 0.79 |
|  | Long-Chain Fatty Acid | palmitate (16:0) | 0.7 | 0.73 |
|  |  | palmitoleate (16:1n7) | 1.03 | 0.56 |
|  |  | 10-heptadecenoate (17:1n7) | 1.36 | 0.56 |
|  |  | stearate (18:0) | 0.89 | 0.76 |
|  |  | 10-nonadecenoate (19:1n9) | 0.72 | 0.66 |
|  |  | arachidate (20:0) | 1.04 | 0.74 |
|  |  | eicosenoate (20:1) | 0.56 | 0.68 |
|  |  | erucate (22:1n9) | 0.61 | 0.55 |
|  |  | oleate/vaccenate (18:1) | 0.31 | 0.49 |
|  | Polyunsaturated Fatty Acid (n3 and n6) | eicosapentaenoate (EPA; 20:5n3) | 0.41 | 0.48 |
|  |  | docosapentaenoate (n3 DPA; 22:5n3) | 0.58 | 0.66 |
|  |  | docosahexaenoate (DHA; 22:6n3) | 0.58 | 0.33 |
|  |  | linoleate (18:2n6) | 0.42 | 0.52 |
|  |  | linolenate [alpha or gamma; (18:3n3 or 6)] | 0.12 | 0.25 |
|  |  | dihomo-linolenate (20:3n3 or n6) | 0.24 | 0.67 |
|  |  | arachidonate (20:4n6) | 0.57 | 0.59 |
|  |  | dihomo-linoleate (20:2n6) | 0.37 | 0.62 |
|  | Short-Chain Fatty Acid | valerate | 1.2 | 2.24 |
|  |  | 3-hydroxybutyrate (BHBA) | 0.81 | 0.73 |
| Carbohydrate | Fructose, Mannose and Galactose Metabolism | fructose | 0.99 | 1.67 |
|  |  | mannitol/sorbitol | 0.76 | 0.81 |
|  |  | mannose | 1.6 | 1.6 |
|  |  | mannose-6-phosphate | 1.84 | 1.54 |
|  |  | galactitol (dulcitol) | 1.79 | 2.02 |
|  | Glycolysis, Gluconeogenesis, and Pyruvate Metabolism | glucose | 1.25 | 1.27 |
|  |  | glucose 6-phosphate | 1.83 | 1.15 |
|  |  | fructose-6-phosphate | 1.6 | 1.01 |
|  |  | Isobar: fructose 1,6-diphosphate, glucose 1,6-diphosphate, myo-inositol 1,4 or 1,3-diphosphate | 1.11 | 0.72 |
|  |  | dihydroxyacetone phosphate (DHAP) | 1.48 | 0.85 |
|  |  | 3-phosphoglycerate | 0.32 | 0.14 |
|  |  | phosphoenolpyruvate (PEP) | 0.22 | 0.04 |
|  |  | pyruvate | 1.15 | 1.29 |
|  |  | lactate | 1.63 | 1.61 |
|  |  | glycerate | 0.72 | 0.43 |
|  | Pentose Phosphate Pathway | 6-phosphogluconate | 1.32 | 1.38 |
|  |  | ribose 5-phosphate | 1.19 | 1.15 |
|  |  | ribose 1-phosphate | 0.56 | 0.53 |
|  |  | ribulose/xylulose 5-phosphate | 1.66 | 2.65 |
| Energy | Glycogen Metabolism | maltotetraose | 0.78 | 0.67 |
|  |  | maltotriose | 0.87 | 0.78 |
|  |  | maltose | 1.19 | 1.02 |
|  | Oxidative Phosphorylation | acetylphosphate | 0.88 | 0.45 |
|  |  | phosphate | 1.02 | 1.21 |
|  | TCA Cycle | citrate | 0.79 | 1.34 |
|  |  | aconitate [cis or trans] | 0.62 | 1.09 |
|  |  | alpha-ketoglutarate | 0.98 | 1.41 |
|  |  | succinylcarnitine (C4-DC) | 1.84 | 2.11 |
|  |  | succinate | 0.79 | 0.84 |
|  |  | fumarate | 1.2 | 1.33 |
|  |  | malate | 1.18 | 1.21 |
|  |  | 2-methylcitrate/homocitrate | 1.84 | 2.39 |
| Amino Acid | Alanine and Aspartate Metabolism | alanine | 1.1 | 1.27 |
|  |  | N-acetylalanine | 1.25 | 1.18 |
|  |  | N-methylalanine | 1.76 | 1.76 |
|  |  | aspartate | 0.95 | 1.07 |
|  |  | N-acetylaspartate (NAA) | 0.38 | 1.12 |
|  |  | asparagine | 0.92 | 1.54 |
|  |  | N-acetylasparagine | 1.19 | 1.23 |
|  | Arginine, ornithine and Proline Metabolism | arginine | 1.07 | 1.31 |
|  |  | argininosuccinate | 1.79 | 1.48 |
|  |  | urea | 0.77 | 0.75 |
|  |  | ornithine | 1.3 | 1.18 |
|  |  | 2-oxoarginine* | 0.81 | 0.75 |
|  |  | citrulline | 1.3 | 1.01 |
|  |  | homoarginine | 1.61 | 1.46 |
|  |  | homocitrulline | 0.87 | 0.98 |
|  |  | proline | 1.19 | 1.39 |
|  |  | dimethylarginine (SDMA + ADMA) | 1.46 | 1.61 |
|  |  | N-acetylarginine | 0.96 | 0.89 |
|  |  | N-delta-acetylornithine | 0.36 | 0.4 |
|  |  | trans-4-hydroxyproline | 0.6 | 0.82 |
|  |  | N-methylproline | 0.5 | 0.5 |
|  |  | argininate* | 1.28 | 0.95 |
|  | Aromatic aminoacids (AAA): tryptophan, tyrosine, phenylalanine and histidine metabolism | phenylalanine | 1.16 | 1.12 |
|  |  | N-acetylphenylalanine | 0.76 | 0.82 |
|  |  | phenyllactate (PLA) | 1.29 | 1.13 |
|  |  | tyrosine | 1.19 | 1.22 |
|  |  | 4-hydroxyphenylpyruvate | 1.41 | 0.87 |
|  |  | 3-(4-hydroxyphenyl)lactate | 1.21 | 1.08 |
|  |  | phenol sulfate | 0.75 | 0.84 |
|  |  | 3-methoxytyrosine | 1.86 | 1.6 |
|  |  | O-methyltyrosine | 1.29 | 1.22 |
|  |  | p-cresol-glucuronide* | 1.65 | 2.26 |
|  |  | 3-hydroxyphenylacetatoylcarnitine | 6.98 | 4.37 |
|  |  | tryptophan | 0.88 | 0.92 |
|  |  | indolepropionate | 0.88 | 1.06 |
|  |  | 3-indoxyl sulfate | 0.85 | 1.03 |
|  |  | indolelactate | 0.8 | 0.72 |
|  |  | kynurenine | 0.8 | 0.85 |
|  |  | kynurenate | 0.57 | 0.71 |
|  |  | N-formylanthranilic acid | 0.75 | 0.79 |
|  |  | tryptophan betaine | 0.67 | 0.6 |
|  |  | C-glycosyltryptophan | 1 | 0.94 |
|  |  | histidine | 0.73 | 1.18 |
|  |  | 1-methylhistidine | 0.65 | 0.75 |
|  |  | trans-urocanate | 1.16 | 0.83 |
|  |  | cis-urocanate | 4.58 | 2.85 |
|  |  | imidazole lactate | 1.72 | 1.92 |
|  |  | carnosine | 1.1 | 1.01 |
|  |  | histamine | 0.49 | 0.56 |
|  |  | 1-methylhistamine | 0.71 | 0.76 |
|  |  | 1-methylimidazoleacetate | 0.74 | 0.87 |
|  |  | histidine methyl ester | 1.46 | 1.73 |
|  | Dipeptide Derivative | N-acetylcarnosine | 1.34 | 1.28 |
|  |  | homocarnosine | 1.09 | 1.12 |
|  |  | anserine | 0.89 | 0.78 |
|  | Glutamate Metabolism | glutamate | 1.2 | 1.33 |
|  |  | glutamine | 1.04 | 1.31 |
|  |  | N-acetylglutamate | 1.82 | 1.45 |
|  |  | N-acetylglutamine | 1.23 | 1.52 |
|  |  | glutamate, gamma-methyl ester | 1.11 | 1.27 |
|  |  | pyroglutamine* | 2.95 | 2.23 |
|  |  | N-acetyl-aspartyl-glutamate (NAAG) | 0.88 | 0.91 |
|  |  | beta-citrylglutamate | 0.57 | 0.85 |
|  | Glutathione metabolism | glutathione, reduced (GSH) | 2.02 | 1.51 |
|  |  | glutathione, oxidized (GSSG) | 1.25 | 1.27 |
|  |  | S-methylglutathione | 2.01 | 1.85 |
|  |  | S-lactoylglutathione | 2.11 | 0.76 |
|  |  | cysteinylglycine | 1.95 | 1.92 |
|  |  | 5-oxoproline | 1.12 | 1.48 |
|  |  | 2-hydroxybutyrate/2-hydroxyisobutyrate | 0.93 | 0.87 |
|  |  | ophthalmate | 1.14 | 1.37 |
|  |  | 4-hydroxy-nonenal-glutathione | 0.11 | 0.22 |
|  | Glycine, Serine and Threonine Metabolism | glycine | 1.2 | 1.23 |
|  |  | N-acetylglycine | 1.06 | 1.05 |
|  |  | sarcosine | 1.78 | 2.33 |
|  |  | dimethylglycine | 1.19 | 1.13 |
|  |  | betaine | 0.93 | 0.91 |
|  |  | serine | 1.29 | 1.76 |
|  |  | N-acetylserine | 1.54 | 1.48 |
|  |  | threonine | 1 | 1.27 |
|  |  | N-acetylthreonine | 1.42 | 1.19 |
|  | Lysine Metabolism | lysine | 1.03 | 1.44 |
|  |  | N6-acetyllysine | 1.73 | 1.54 |
|  |  | N6,N6,N6-trimethyllysine | 1.05 | 1.07 |
|  |  | 5-(galactosylhydroxy)-L-lysine | 0.78 | 0.95 |
|  |  | saccharopine | 1 | 1.07 |
|  |  | 2-aminoadipate | 1.26 | 1.59 |
|  |  | glutarate (pentanedioate) | 1.13 | 1.26 |
|  |  | glutarylcarnitine (C5-DC) | 1.49 | 1.7 |
|  |  | pipecolate | 0.47 | 0.48 |
|  |  | 6-oxopiperidine-2-carboxylate | 1.02 | 1.15 |
|  |  | 5-aminovalerate | 1.06 | 0.89 |
|  | Methionine, Cysteine, SAM and Taurine Metabolism | methionine | 1 | 1.03 |
|  |  | N-acetylmethionine | 1.45 | 1.35 |
|  |  | N-formylmethionine | 1.64 | 1.48 |
|  |  | S-methylmethionine | 1.21 | 1.32 |
|  |  | methionine sulfone | 0.61 | 0.66 |
|  |  | methionine sulfoxide | 0.75 | 0.82 |
|  |  | N-acetylmethionine sulfoxide | 1.53 | 0.89 |
|  |  | S-adenosylmethionine (SAM) | 1.06 | 0.97 |
|  |  | S-adenosylhomocysteine (SAH) | 1.11 | 1.02 |
|  |  | cysteine | 1.16 | 0.85 |
|  |  | S-methylcysteine | 1.67 | 1.57 |
|  |  | S-methylcysteine sulfoxide | 2.41 | 2.44 |
|  |  | hypotaurine | 0.9 | 0.79 |
|  |  | taurine | 1.03 | 1.1 |
|  |  | N-acetyltaurine | 0.6 | 4.15 |
|  |  | taurocyamine | 3.22 | 2.73 |
|  | Proteinogenic BCAA's isoleucine, leucine and valine metabolism | leucine | 0.85 | 0.87 |
|  |  | N-acetylleucine | 0.67 | 0.9 |
|  |  | 4-methyl-2-oxopentanoate | 0.67 | 0.57 |
|  |  | alpha-hydroxyisocaproate | 0.86 | 0.75 |
|  |  | isovalerylglycine | 0.67 | 0.56 |
|  |  | isovalerylcarnitine (C5) | 0.65 | 0.58 |
|  |  | beta-hydroxyisovalerate | 0.62 | 0.82 |
|  |  | beta-hydroxyisovaleroylcarnitine | 1.57 | 1.35 |
|  |  | 3-methylglutarylcarnitine (2) | 1.98 | 2.04 |
|  |  | isoleucine | 1.03 | 1.05 |
|  |  | N-acetylisoleucine | 0.91 | 1.11 |
|  |  | 3-methyl-2-oxovalerate | 0.76 | 0.62 |
|  |  | alpha-hydroxyisovalerate | 0.89 | 0.84 |
|  |  | 2-methylbutyrylcarnitine (C5) | 0.77 | 0.65 |
|  |  | tiglylcarnitine (C5:1-DC) | 1.04 | 0.95 |
|  |  | ethylmalonate | 1.1 | 1.37 |
|  |  | methylsuccinate | 0.94 | 1.19 |
|  |  | valine | 0.79 | 0.74 |
|  |  | 3-methyl-2-oxobutyrate | 0.63 | 0.64 |
|  |  | 2-hydroxy-3-methylvalerate | 1.27 | 1.07 |
|  |  | isobutyrylcarnitine (C4) | 0.44 | 0.52 |
|  |  | isobutyrylglycine | 0.66 | 0.63 |
|  |  | 3-hydroxyisobutyrate | 0.74 | 0.63 |
|  |  | glycylleucine | 2.43 | 2.04 |
|  |  | glycylvaline | 1.72 | 1.23 |
|  |  | leucylglycine | 1.54 | 0.99 |
|  |  | phenylalanylalanine | 2.24 | 0.94 |
|  |  | prolylglycine | 1.27 | 1.21 |
|  |  | valylleucine | 2.56 | 2.03 |
| Nucleotide | Purine Metabolism, (Hypo)Xanthine/Inosine containing | inosine 5'-monophosphate (IMP) |  | 0.53 |
|  |  | inosine |  | 0.63 |
|  |  | N1-methylinosine |  | 0.27 |
|  | Purine Metabolism, Adenine containing | adenosine 5'-monophosphate (AMP) |  | 0.28 |
|  |  | adenosine |  | 0.19 |
|  | Purine Metabolism, Guanine containing | guanosine 5'- monophosphate (5'-GMP) |  | 0.13 |
|  |  | guanosine |  | 0.33 |
|  | Pyrimidine Metabolism, Cytidine containing | cytidine 5'-monophosphate (5'-CMP) |  | 0.62 |
|  |  | cytidine |  | 0.30 |
|  |  | cytidine diphosphate |  | 0.70 |
|  | Pyrimidine Metabolism, Thymine containing | 3-aminoisobutyrate |  | 1.58 |
|  | Pyrimidine Metabolism, Uracil containing | uridine |  | 0.69 |
| Xenobiotic | | 4-ethylphenylsulfate |  | 0.44 |
|  |  | 4-methylcatechol sulfate |  | 0.34 |
|  |  | benzoylcarnitine* |  | 1.96 |
|  |  | 4-acetylphenol sulfate |  | 0.28 |
|  |  | N-glycolylneuraminate |  | 2.65 |
|  |  | erythritol |  | 5.82 |
|  |  | methyl glucopyranoside (alpha + beta) |  | 2.70 |
|  |  | umbelliferone sulfate |  | 0.07 |
